# Water Extract of Desalted *Salicornia europaea* Inhibits RANKL-Induced Osteoclast Differentiation and Prevents Bone Loss in Ovariectomized Mice

**DOI:** 10.3390/nu15234968

**Published:** 2023-11-30

**Authors:** Ah-Ra Jang, Yun-Ji Lee, Dong-Yeon Kim, Tae-Sung Lee, Do-Hyeon Jung, Yeong-Jun Kim, In-Su Seo, Jae-Hun Ahn, Eun-Jung Song, Jisu Oh, Aoding Li, SiHoon Song, Hyung-Sik Kim, Min-Jung Kang, Yoojin Seo, Jeong-Yong Cho, Jong-Hwan Park

**Affiliations:** 1 Laboratory Animal Medicine, College of Veterinary Medicine, Animal Medical Institute, Chonnam National University, Gwangju 61186, Republic of Korea; 2 NODCURE, Inc., 77 Yongbong-ro, Buk-gu, Gwangju 61186, Republic of Korea; 3 Department of Food Science and Technology, Chonnam National University, Gwangju 61186, Republic of Korea; 4 Department of Oral Biochemistry, Dental and Life Science Institute, School of Dentistry, Pusan National University, Yangsan 50612, Republic of Korea; 5 Department of Life Science in Dentistry, School of Dentistry, Pusan National University, Yangsan 50612, Republic of Korea; 6 Education and Research Team for Life Science on Dentistry, Pusan National University, Yangsan 50612, Republic of Korea

**Keywords:** *Salicornia europaea*, dicaffeoylquinic acids, bone loss, osteoclast, reactive oxygen

## Abstract

Osteoporosis, which is often associated with increased osteoclast activity due to menopause or aging, was the main focus of this study. We investigated the inhibitory effects of water extract of desalted *Salicornia europaea* L. (WSE) on osteoclast differentiation and bone loss in ovariectomized mice. Our findings revealed that WSE effectively inhibited RANKL-induced osteoclast differentiation, as demonstrated by TRAP staining, and also suppressed bone resorption and F-actin ring formation in a dose-dependent manner. The expression levels of genes related to osteoclast differentiation, including NFATc1, ACP5, Ctsk, and DCSTAMP, were downregulated by WSE. Oral administration of WSE improved bone density and structural parameters in ovariectomized mice. Dicaffeoylquinic acids (DCQAs) and saponins were detected in WSE, with 3,4-DCQA, 3,5-DCQA, and 4,5-DCQA being isolated and identified. All tested DCQAs, including the aforementioned types, inhibited osteoclast differentiation, bone resorption, and the expression of osteoclast-related genes. Furthermore, WSE and DCQAs reduced ROS production mediated by RANKL. These results indicate the potential of WSE and its components, DCQAs, as preventive or therapeutic agents against osteoporosis and related conditions.

## 1. Introduction

The process of bone remodeling consists of the resorption of bone by osteoclasts and the generation of new bone by osteoblasts. Maintaining a balance between these two types of cells is crucial for preserving bone mass and mineral metabolism. An imbalance between the bone resorption of osteoclast and the bone formation of osteoblast can lead to bone-related conditions, including osteoporosis and rheumatoid arthritis [1]. Postmenopausal osteoporosis, a common metabolic bone disorder in older women, is associated with decreased hormone levels [2]. Estrogen deficiency leads to osteoclastic bone resorption and bone loss, ultimately causing osteoporosis [3]. Hence, inhibiting osteoclast differentiation is considered the primary therapeutic approach for treating osteoporosis.

Osteoclasts, which develop in the mononuclear cell/macrophage hematopoietic lineage, exhibit a multinucleated morphology. The differentiation and activity of osteoclasts depend on two essential cytokines: the receptor activator of nuclear factor-kappa B (NF-κB) ligand (RANKL), a differentiation factor, and the macrophage-colony stimulating factor (M-CSF), a survival factor [4]. When RANKL binds to RANK, TNF receptor-associated factor 6 (TRAF6) is recruited, leading to the activation of downstream signaling factors such as NF-κB and mitogen-activated protein kinases (MAPKs). Subsequently, the nuclear factor of activated T cells c1 (NFATc1), a key regulator of osteoclast differentiation, is activated and regulates several osteoclast-specific genes including ACP5, Ctsk, and DCSTAMP [5].

*Salicornia europaea* L. (SE), also known as *Salicornia herbacea* L., is an annual halophyte that grows in salt marshes. It belongs to the Amaranthaceae family and is distributed along the west coast of the Korean peninsula. Its common name is glasswort, and it is commonly referred to as hamcho in Korea. This plant has traditionally been used in salads, fermented foods, and other dishes [6]. It is also used as a folk medicine to treat various diseases such as headaches and scurvy [7]. Additionally, in our country, it is commercially available as a health supplement. Recent studies have suggested that SE has beneficial properties due to its antioxidant, anti-inflammatory, anti-neuroinflammatory, anti-amnesic, anti-diabetic, and anti-hyperlipidemic effects [8,9,10]. Moreover, SE has been considered to potentially have anti-osteoporotic effects based on its ability to inhibit adipogenesis and promote osteoblast differentiation [11]. Since SE grows under high-salt conditions, it contains abundant bioactive phytochemicals, such as flavonoids and saponins, to overcome salt stress [9,12]. Dicaffeoylquinic acids (DCQAs) and two flavonoid glycosides (isorhamnetin 3-O-β-D-glucoside and quercetin 3-O-β-D-glucoside) isolated from SE have potential as anti-metastatic and anti-cancer agents that inhibit MMP9 and/or MMP2 activity in fibrosarcoma HT-1080 cells [13]. However, to the best of our knowledge, studies on the osteoclast inhibitory mechanism and the anti-osteoporotic effect of SE and its constituents have not been reported to date.

Natural sources extracted by water, ethanol, or their mixtures have been increasingly utilized as food additives in various foods and in preventive or therapeutic agents, because residual solvents pose a potential risk to human health [14]. Among the extraction methods, the hot water extraction method is considered the most useful, and it is easy to perform [15]. In this study, we compared the inhibitory effects of hot water and ethanol extracts of *S. europaea* on osteoclast differentiation. As the efficacies were similar, we ultimately selected the hot water extract after considering the ease of its industrial scale-up. Our focus in this study is on the inhibitory effects of the water extract of desalted *S. europaea* on RANKL-induced osteoclast differentiation and bone loss in ovariectomized (OVX) mice, as well as the identification of their related bioactive compounds.

## 2. Materials and Methods

### 2.1. Sample Preparation and Isolation of Three DCQAs

The dried aerial parts of SE were obtained from Dasarang Co., Ltd., located in Shinan County, South Korea. Ground samples (1.3 kg) were soaked in 52 L of distilled water at room temperature for 1 h. After draining, the residues were extracted with 19.5 L of hot water 95 °C for 5 h. The hot water extracts were then filtered through a 140-mesh filter (Whatman, Maidstone, UK) and concentrated at 60 °C under vacuum conditions until reaching a solid content of 15 °Brix. These concentrates were spray-dried at operating conditions including an inlet air temperature (190 °C) and an outlet air temperature (60 °C) to obtain WSE. Three DCQAs were purified and isolated from the WSE using ODS column chromatography, and their identification was confirmed through MS and NMR analyses (Supplementary Materials). The MS and NMR spectroscopic data are provided in Supplementary Materials.

### 2.2. Osteoclast Differentiation 

Bone marrow cells were isolated from the femurs of 8-week-old male mice (Koatech, Pyeongtaek, Republic of Korea) and cultured in α-MEM containing 10% FBS (Gibco, Grand Island, NY, USA), 1% penicillin/streptomycin, and recombinant M-CSF (25 ng/mL; Miltenyi Biotec, Bergisch Gladbach, Germany) in a 5%-CO incubator at 37 °C. After 3 days, BMDMs were seeded and incubated in the presence of recombinant RANKL (100 ng/mL; Peprotech, Cranbury, NJ, USA) for 3 days, with daily medium changes.

### 2.3. TRAP Staining

Osteoclast formation was assessed using a TRAP staining kit (Kamiya Biomedical Company, Tukwila, WA, USA) following the manufacturer’s instructions. The number of TRAP-positive cells with three or more nuclei was determined by examining them under a light microscope (Leica, Germany).

### 2.4. F-Actin Ring Formation Staining

BMDMs were plated on cover glass and induced to differentiate into osteoclasts. The cells were fixed using 4% formaldehyde and permeabilized using 0.1% Triton X-100 (Sigma-Aldrich, St. Louis, MO, USA). They were then stained with Alexa Fluor 594-phalloidin (Invitrogen, Carlsbad, CA, USA) for 2 h. Subsequently, the cells were mounted onto slides, and the nuclei were counterstained with 4′,6-diamidino-2-phenylindole (Invitrogen). Images were observed using a fluorescence microscope.

### 2.5. Bone Resorption Assay

BMDMs were cultured on an Osteo Assay Surface multiple-well plate (Corning Costar, Corning, NY, USA). After 7 days, the RANKL-stimulated BMDMs were treated with 20% SDS for 10 min to remove the cells. The absorbed areas on the discs were examined using a microscope and quantified using ImageJ (version: 1.53e) software (National Institutes of Health, Bethesda, MD, USA).

### 2.6. Quantitative Reverse Transciption PCR (RT-qPCR)

Total RNA was isolated from cells using an Easy-BLUE Total RNA Extraction Kit (Intron Biotechnology, Seongnam, Republic of Korea). Complementary DNA was generated using ReverTra Ace qPCR RT Master Mix (TOYOBO Bio-Technology, Osaka, Japan), following the manufacturer’s instructions. The primers used for qPCR are listed in Table 1. Quantitative PCR was performed using QGreen 2× SybrGreen qPCR Master Mix on a CFX Connect Real-time PCR Detection System (Bio-Rad, Hercules, CA, USA). β-actin, a housekeeping gene, was used as an internal control for the normalization of target gene expression. Data were analyzed using the 2^−∆∆Ct^ method, where ∆∆Ct = (Ct_target gene_ − Ct_β-actin_)_target sample_ − (Ct_target gene_ − Ct_β-actin_)_control sample_.

### 2.7. Western Blot

Cells were lysed in lysis buffer supplemented with a protease inhibitor (Roche, Mannheim, Germany) and phosphatase inhibitor (Sigma-Aldrich, St. Louis, MO, USA). Blots were then probed with primary antibodies (diluted 1:1000 in TBS containing 0.05% Tween-20) against IκB-α, phospho-form of p65, JNK, p38, and ERK (Cell Signaling Technology, Beverly, MA, USA), and β-actin (Santa Cruz Biotechnology, Dallas, TX, USA). The secondary antibodies used were HRP-labelled anti-rabbit or anti-mouse antibodies diluted 10,000-fold in 5% skim milk. The proteins were detected using Clarity Western ECL Substrate (Bio-Rad, Hercules, CA, USA), and the protein band densities were quantified using ImageJ (version: 1.53e).

### 2.8. Intracellular ROS Detection

The detection of intracellular ROS was conducted using a previously established method [16]. In brief, BMDMs were stimulated with RANKL with or without WSE, DCQA fraction, and three compounds at the indicated concentrations for 24 h. Cells were washed and then incubated with 2′, 7′-dichlorofluorescin diacetate (Sigma-Aldrich, St. Louis, MO, USA) for 30 min at 37 °C. The oxidative conversion was detected using a fluorescence microscope. This experiment involved cell counting through repeated trials to quantify the number of ROS-positive cells.

### 2.9. Animal Experiments

Seven-week-old female C57BL/6 mice were purchased from Koatech and acclimated for one week. The mice were then divided into two groups: the sham group (*n* = 7), which underwent sham surgery, and the OVX group (*n* = 31), which underwent bilateral ovariectomy. After a ten-day recovery period, the ovariectomized mice were randomly assigned to four groups: the vehicle control group (*n* = 7) and three groups treated with different doses of WSE (40, 80, 160 mg/kg, *n* = 8 each). The vehicle (PBS) and WSE were administrated by gavage six times per week for twelve weeks. At the end of the study, all mice were euthanized, and their femurs were used for micro-computed tomography (μCT). All animal studies conducted were approved by the Institutional Animal Care and Use Committee of Chonnam National University (Approval No. 2021-111).

### 2.10. Micro-CT Analysis

The femurs were analyzed using high-resolution μCT (Sky-Scan 1172TM, Skyscan, Kontich, Belgium). To determine the femoral morphometry, bone alterations were quantified by analyzing bone mineral density (BMD), bone volume per tissue volume (BV/TV), trabecular thickness (Tb.Th), trabecular spacing (Tb.Sp), and trabecular number (Tb.n) using data analysis software (CTAn).

### 2.11. Statistical Analysis

All in vitro experiments were independently repeated twice or three times, and one representative result was presented. The statistical significance of the intergroup differences was determined using one-way analysis of variance (ANOVA), followed by the Dunnett test. All statistical analyses were performed using the GraphPad Prism 8 (GraphPad Software, San Diego, CA, USA). A *p*-value < 0.05 was considered to indicate a statistically significant difference.

## 3. Result

### 3.1. WSE Inhibited RANKL-Induced Osteoclast Differentiation and Bone Resorption

**Figure 1:**
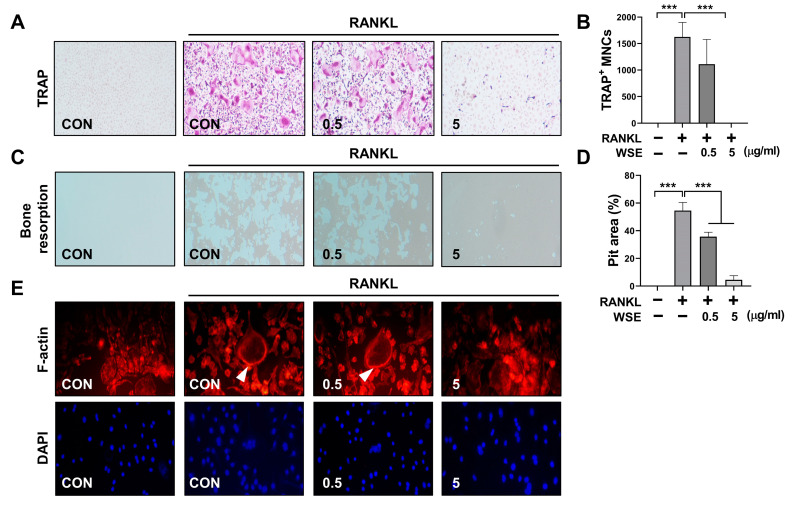
WSE suppressed RANKL-induced osteoclast differentiation. BMDMs were pretreated with WSE (0.5 and 5 μg/mL) for 2 h and subsequently stimulated with RANKL for 3 days. RANKL-induced osteoclasts were fixed and stained to detect TRAP activity (**A**). TRAP-positive cells were counted (**B**). Representative images of bone resorption pits were observed (**C**), and resorption pits were quantified using ImageJ software (**D**). RANKL-induced F-actin rings were stained with Alexa Fluor 594-phalloidin and visualized; white arrowheads: all mOC showed an actin ring (**E**). The results are presented as the mean ± SD. *** *p* < 0.001.

The formation of TRAP-positive cells was completely inhibited by WSE at a dose of 5 μg/mL (Figure 1A,B). WSE also induced a dose-dependent decrease in the area percentage of absorption pits (Figure 1C,D). Osteoclasts form F-actin rings to reorganize the actin cytoskeleton and create resorption cavities on the bone surface [17]. Therefore, we investigated the impact of WSE on F-actin ring formation. WSE treatment inhibited F-actin ring formation caused by RANKL (Figure 1E). Next, to investigate the potential involvement of WSE in osteoblast differentiation, we induced the differentiation of hUCB-MSCs into osteogenic cells. WSE had no effect on osteoblast differentiation (Figure S21A–D). These results suggest that WSE exerts an inhibitory effect on osteoclast differentiation and bone resorption induced by RANKL.

### 3.2. WSE Suppressed the Gene Expression Associated with RANKL-Induced Osteoclastogenesis in BMDMs

**Figure 2:**
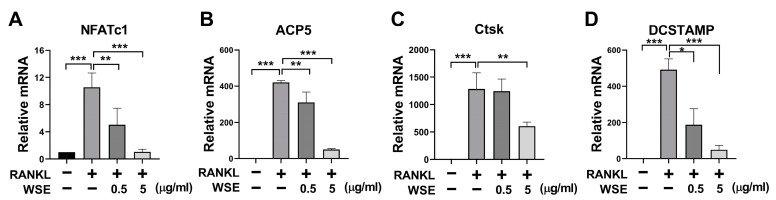
WSE inhibited RANKL-induced osteoclast-specific gene expression. The mRNA expression levels of *NFATc1* (**A**), *ACP5* (**B**), *Ctsk* (**C**), and *DCSTAMP* (**D**) were quantified using qPCR. β-actin was used as the internal control. The results are presented as the mean ± SD. * *p* < 0.05; ** *p* < 0.01; *** *p* < 0.001.

NFATc1 is expressed during intermediate or late stages of osteoclast differentiation and regulates the transcription of osteoclast-specific genes [5]. To confirm the inhibitory effect of WSE on osteoclast differentiation induced by RANKL, we further analyzed the expression of osteoclast-specific genes through qPCR. RANKL significantly increased the mRNA levels of *NFATc1, ACP5, Ctsk*, and *DCSTAMP*, which were downregulated using WSE treatment in a dose-dependent manner (Figure 2A–D). 

### 3.3. Oral Administration of WSE Attenuated OVX-Induced Bone Loss in Mice

**Figure 3:**
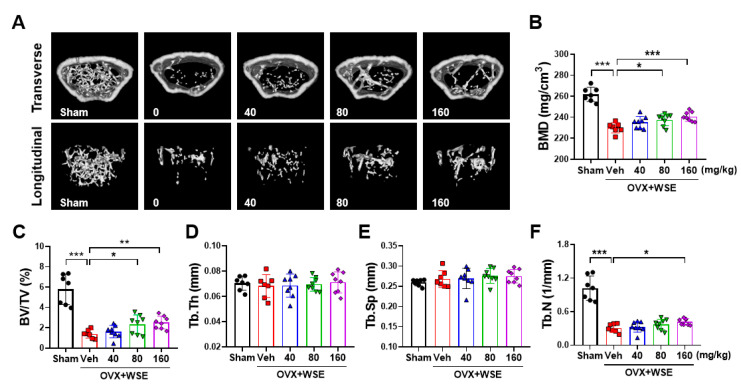
WSE prevented OVX-induced bone loss in mice. Representative μCT images of mouse femurs (**A**). Analyses of BMD (**B**), BV/TV (**C**), Tb.Th (**D**), Tb.Sp (**E**), and Tb.n (**F**) were conducted using μCT. The results are presented as the mean ± SD. * *p* < 0.05; ** *p* < 0.01; *** *p* < 0.001.

We conducted further investigations to determine whether the administration of WSE could reduce bone loss in OVX mice. The body weight of the OVX mice was higher compared to the sham controls. However, upon WSE administration, the body weight significantly decreased in a dose-dependent manner (Figure S1A–C). Additionally, the increase in abdominal fat associated with the OVX surgery was significantly reduced in the group that received 160 mg/kg WSE compared to the OVX controls (Figure S1E). Quantitative morphological analysis revealed that the OVX group had significantly lower BMD compared to the sham group (Figure 3A,B). These changes were partially ameliorated via the oral administration of WSE in a dose-dependent manner (Figure 3A,B). WSE administration increased BV/TV and Tb.n, which were decreased by OVX, while Tb.Th and Tb.Sp did not return to baseline levels (Figure 3C–F). These findings suggest that the oral administration of WSE may help prevent bone loss in postmenopausal women.

### 3.4. Identification of Three DCQAs in WSE

**Figure 4:**
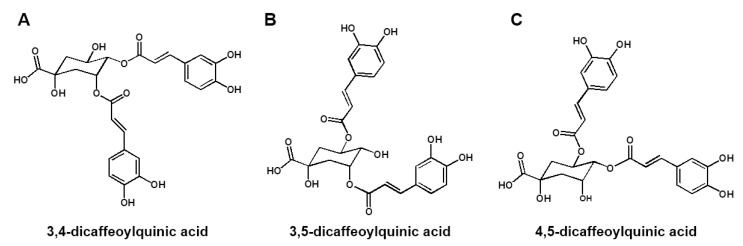
Structures of three DCQAs isolated from WSE. Structures of 3,4-DCQA (**A**), 3,5-DCQA (**B**) and 4,5-DCQA (**C**) isolated from WSE.

We performed analyses using UPLC-ESI-Q-TOF MS with WSE samples to obtain a metabolite profile. Thirty-three compounds, including DCQAs, flavonoids, and triterpene glycosides, were detected on the ion chromatograms (Figure S2 and Table S1) [18,19,20]. Among these compounds, DCQAs and triterpene glycosides were considered the main compounds in WSE. To determine the accurate structures of the metabolites, we attempted to purify and isolate compounds from the WSE. Through ODS column chromatography, we successfully isolated three DCQAs as white amorphous powder from the WSE sample. The high purities (>95%) of the three isolated DCQAs were confirmed by their MS and 1H NMR spectra (Figures S3−S7, S11, S12, S16 and S17), and their HR-ESI-MS data confirmed that they had the same molecular weight (516) and molecular formula (C25H24O12) (Table S2). The MS results were supported by their 13C NMR spectra, which showed a total of twenty-five carbon signals, including three carbonyl carbons at δ 178.6–169.6 and one oxygenated quaternary carbon of quinic acid at δ 71.5–75.4 (C-1) (Table S4). In addition, the 1H NMR spectra of the three DCQAs confirmed the presence of DCQA corresponding to the proton signals of two caffeic acids at δ 6.26–7.62 (H-2′–H-8′ and H-2″–H-8″) and one quinic acid at δ 2.13–5.67 (H-2–H-6) (Table S3). However, the chemical shifts of three oxygenated proton signals at δ 4.32–5.67 in quinic acid differed significantly among the three DCQAs. The connections between caffeic acid and quinic acid were confirmed through the 2D-NMR experiments, including 1H-1H COSY, HSQC, and HMBC (Figures S8–S10, S13–S15 and S18–S20). Finally, the three DCQAs were identified as 3,4-DCQA, 3,5-DCQA, and 4,5-DCQA by comparing them with previously reported MS and NMR results (Figure 4A–C) [18,19,21].

### 3.5. DCQAs Extracted from WSE Inhibited RANKL-Induced Osteoclast Differentiation

**Figure 5:**
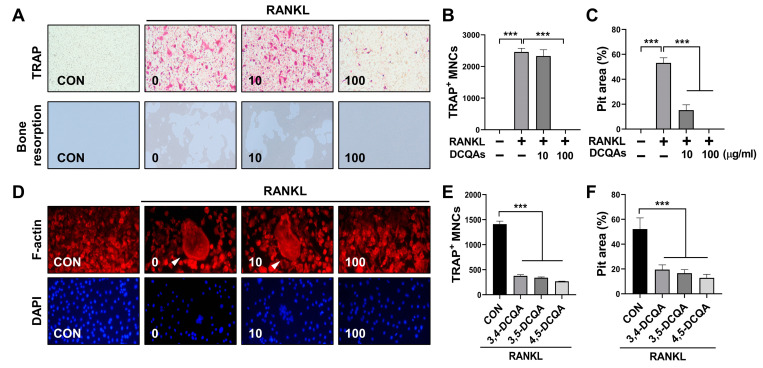
Three DCQA derivatives from WSE suppressed RANKL-induced osteoclast differentiation. BMDMs were pretreated with DCQA fractions (10 and 100 μg/mL) for 2 h and subsequently stimulated with RANKL for 3 days. Osteoclast differentiation and osteoclastic bone resorption induced by RANKL were imaged (**A**). TRAP-positive cells were counted (**B**). Quantification of the resorption pits was performed using ImageJ software (**C**). RANKL-induced F-actin rings were stained with Alexa Fluor 594-phalloidin and imaged; white arrowheads: all mOC showed an actin ring (**D**). BMDMs were pretreated with three types of DCQAs (10 μΜ) for 2 h and subsequently stimulated with RANKL for 3 days. TRAP-positive cells were counted (**E**). Osteoclastic bone resorption function was quantified using ImageJ software (**F**). The results are presented as the mean ± SD. *** *p* < 0.001.

Previous studies have shown that 3,5-DCQA isolated from *Chrysanthemum zawadskii* var. *latilobum* has an inhibitory effect on RANKL-induced osteoclastogenesis [22]. Therefore, we aimed to determine whether a fraction containing DCQAs and chemical compounds isolated from the fraction possesses anti-osteoclastogenic properties. TRAP-positive cells were eliminated after treatment with DCQAs at a dose of 100 μg/mL (Figure 5A,B). Bone resorption induced by RANKL was also suppressed via the DCQA fraction treatment in a dose-dependent manner (Figure 5A–C). Moreover, the DCQA fraction treatment reduced F-actin ring formation by RANKL in a dose-dependent manner (Figure 5D). We further investigated the inhibitory effect of individual compounds present in the fraction. The three DCQAs reduced the number of TRAP-positive cells and the size of the pit area formed by RANKL (Figure S22A,B and Figure 5E,F). 

**Figure 6:**
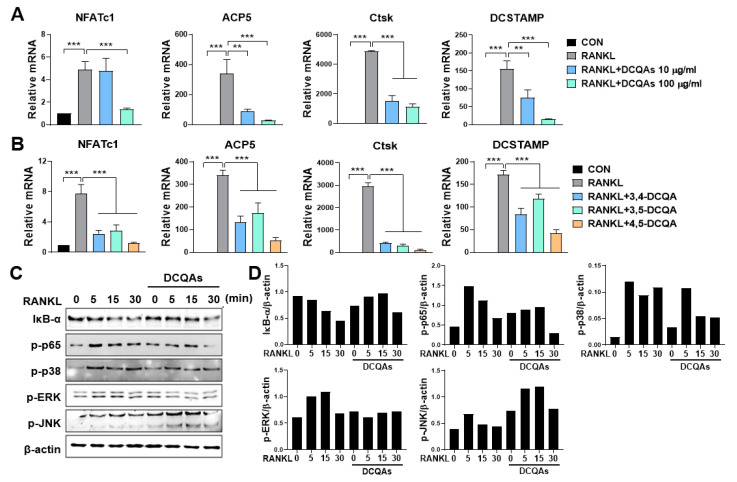
Three DCQA derivatives from WSE regulated RANKL-induced osteoclastogenesis by influencing osteoclast-specific gene expression and inhibiting NF-κB and MAPKs. BMDMs were pretreated with DCQA fractions (10 and 100 μg/mL) and three constituent DCQAs (10 μΜ) for 2 h and subsequently stimulated with RANKL for 3 days. Regulation of gene expression levels of NFATc1, ACP5, Ctsk, and DCSTAMP via the DCQA fractions and the three constituent DCQAs (**A**,**B**) was determined using qPCR. β-actin was used as the internal control. Western blotting was used to analyze the expression of IκB-α degradation, as well as the phosphorylation of p65, p38, ERK, and JNK in RANKL-stimulated BMDMs (**C**). Antibody against β-actin was used to confirm the loading doses. Quantification of protein levels was performed using ImageJ software (**D**). The results are presented as the mean ± SD. ** *p* < 0.01; *** *p* < 0.001.

Next, we investigated the effect of DCQA on the mRNA expression of osteoclast-specific genes. As expected, fractions containing DCQAs inhibited the RANKL-induced expression of all tested genes in a dose-dependent manner (Figure 6A). The three DCQAs also effectively inhibited the expression of the corresponding genes, with 4,5-DCQA showing the highest inhibitory effect. We also examined whether the DCQA-containing fraction influenced the RANKL-induced activation of NF-κB and MAPKs. Western blot analysis showed that RANKL treatment enhanced the activation of NF-κB and MAPKs, including ERK, p38, and JNK (Figure 6C,D). The phosphorylation of p65, ERK, and p38, and the degradation of IκB-α were effectively inhibited by treatment with the DCQA fraction, although JNK phosphorylation was not affected (Figure 6C,D). These experiments were repeated and validated (Figure S23A,B). These findings suggest that DCQAs may be the essential compounds of WSE associated with the inhibition of osteoclast differentiation. 

### 3.6. WSE and DCQAs Reduced ROS Production Induced by RANKL

**Figure 7:**
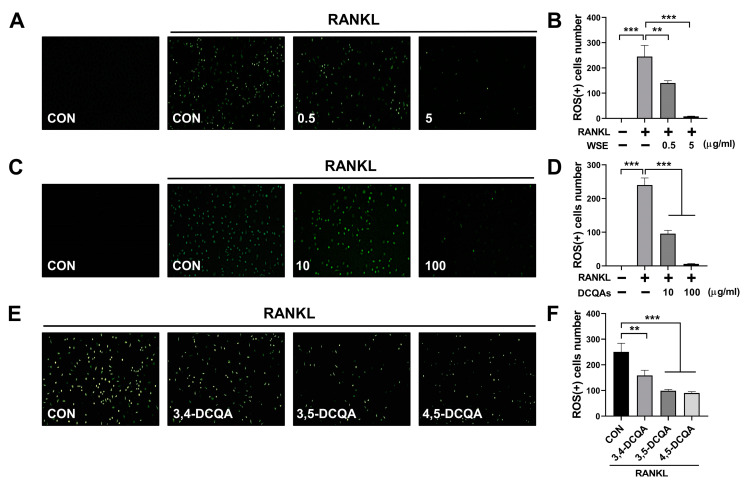
WSE and its compounds suppressed RANKL-induced ROS production. BMDMs were pretreated with WSE (0.5 and 5 μg/mL), DCQA fractions (10 and 100 μg/mL), and three constituent DCQAs (10 μM) for 2 h and subsequently stimulated with RANKL. After 24 h, cells were incubated with DCFDA (2′, 7′-dichlorofluorescin diacetate) and analyzed using fluorescence microscopy (**A**,**C**,**E**). The number of ROS-positive cells was counted in each well (**B**,**D**,**F**). The results are presented as the mean ± SD. ** *p* < 0.01; *** *p* < 0.001.

ROS promote osteoclast differentiation and play a role in the early stages of osteoclast activation. RANKL-induced intracellular ROS production is considered an upstream factor that regulates osteoclast differentiation and activation [23,24]. Accordingly, we investigated whether WSE and DCQAs have an effect on RANKL-induced intracellular ROS production. We found that WSE dose-dependently suppressed the RANKL-induced ROS production and almost completely inhibited it at a dose of 5 μg/mL (Figure 7A,B). The number of ROS-positive cells was also reduced via treatment with the DCQA fraction in a dose-dependent manner (Figure 7C,D). All three compounds tested showed inhibitory effects on RANKL-induced ROS formation (Figure 7E,F). These results suggest that WSE and DCQAs may inhibit RANKL-induced osteoclast differentiation by scavenging ROS.

## 4. Discussion

Bone homeostasis is maintained through the balance of osteoclasts and osteoblasts. This balance can be disrupted by various processes and conditions, such as menopause, aging, and chronic inflammation [1,25]. These disruptions can lead to bone-related diseases, including osteoporosis. In postmenopausal women, osteoporosis is caused by accelerated osteoclast differentiation due to estrogen deficiency [2]. Many studies have found that excessive differentiation and activity of osteoclasts are primarily associated with bone loss and contribute to the progression of metabolic bone diseases [26]. Therefore, inhibiting the differentiation and activation of osteoclasts is considered the most effective approach to prevent and treat osteoporosis.

SE and its bioactive constituents are known to have various health-promoting properties [9,12]. Treatment with the SE extract inhibited adipogenesis and promoted osteoblastogenesis in an in vitro study using 3T3-L1 pre-adipocytes and MC3T3-El pre-osteoblasts [11]. This suggests that the SE extract could potentially be developed as a new preventive or therapeutic agent for osteoporosis, as increased adipogenesis and a lack of osteoblastogenesis contribute to osteoporosis and bone loss. In our present study, we observed that WSE suppressed osteoclast differentiation and bone resorption activity. Additionally, WSE treatment dose-dependently reduced the mRNA expression of osteoclastogenesis-related genes that were upregulated by RANKL. In an in vivo study using OVX mice, oral administration of WSE improved BMD, BV/TV, and Tb.n. These results indicate that WSE administration may help alleviate bone loss caused by estrogen deficiency by inhibiting osteoclastogenesis. A previous study showed that PSTP-3,5-Me specifically inhibits osteoclast differentiation without affecting osteoblast differentiation [27]. Similarly, we observed that WSE selectively inhibits osteoclast differentiation, regardless of its impact on osteoblast differentiation. Karadeniz et al. [11] demonstrated that *S. herbacea* promotes osteoblastogenesis in MC3T3-E1 cells. However, in their study, *S. herbacea* was extracted using dichloromethane and applied to cells at concentrations of 10, 50, and 100 μg/mL, which were higher doses than those in our study even considering desalted SE. Moreover, its effect on osteoclastogenesis was not tested in the study. Further study is required to determine the differences in the main components based on the extraction method, comparing dichloromethane and hot water. SE contains various bioactive constituents such as caffeoylquinic acids, flavonoids, triterpenoid saponins, and pentadecyl ferulate. These compounds play a role in bone remodeling by promoting osteoblastogenesis or suppressing adipogenesis and osteoclastogenesis [12,13,20,28,29]. In the present study, we analyzed the metabolite profile of WSE and identified DCQAs and triterpene glycosides as the main compounds. Additionally, we isolated three types of DCQAs and identified them as 3,4-DCQA, 3,5-DCQA, and 4,5-DCQA by comparing them with previous MS and NMR results [18,19,21]. Treatment with a fraction containing DCQAs, as well as with the three constituent DCQAs, suppressed RANKL-induced osteoclast differentiation and osteoclastic bone resorption. Additionally, the RANKL-induced expression of osteoclastogenesis-related genes was also reduced via treatment with the fraction containing DCQAs, as well as with the three constituent DCQAs, suggesting that DCQAs may be the main components of WSE that inhibit osteoclastogenesis. However, this study is limited by the fact that the physiological activity of triterpene glycosides, another key component of WSE, has not been elucidated. In further studies, we aim to determine the inhibitory effect of triterpene glycosides on RANKL-induced osteoclast differentiation and the preventive effect of triterpene glycosides on bone disease in an OVX-induced osteoporosis animal model.

The binding of RANKL to the RANK receptor leads to the recruitment of TRAF6 and subsequently activates NF-κB and MAPKs responsible for osteoclast differentiation and activation. Both NF-κB and MAPKs can control the expression of NFATc1, a master transcriptional regulator of genes related to osteoclast differentiation [4,5]. In this study, treatment with the DCQA fraction suppressed the activation of NF-κB, p38 and ERK MAPKs. It is likely that bioactive components in WSE, including DCQAs, regulate the upstream signaling (e.g., TRAF6) of NF-κB and MAPKs in osteoclast precursors. Additionally, RANKL can induce the production of ROS in osteoclast precursors through TRAF6-mediated pathways and mediate RANKL-induced osteoclastogenesis by regulating NF-κB and MAPKs activation [24]. Therefore, the search for natural products with antioxidant efficacy can contribute to the prevention of bone disease. In the present study, a fraction containing DCQAs, three constituent DCQA compounds, and WSE was found to inhibit RANKL-induced ROS production. SE is known to contain various anti-oxidative compounds, such as tungtungmadic acid, quercetin, chlorogenic acid, and caffeoylquinic acid [12,18]. A previous study reported that four new DCQA derivatives isolated from SE exert anti-oxidative activity [18]. In addition, triterpenoid saponins isolated from *S. herbacea* have been reported to exhibit antioxidant activity. It is suggested that triterpenoid glycosides isolated from WSE may also possess antioxidant activity. Taken together, these findings indicate that WSE and its bioactive DCQAs may inhibit RANKL-induced osteoclast differentiation by suppressing RANKL-induced ROS production.

## 5. Conclusions

In conclusion, our results revealed that WSE suppresses the differentiation of osteoclasts and its oral administration prevents bone loss in OVX mice. OVX animals are commonly used as an animal model in the study of the prevention and treatment of postmenopausal osteoporosis [30]. WSE was found to be effective in preventing bone loss in OVX mouse models. Therefore, WSE supplementation has the potential to be a valuable preventive measure against bone loss resulting from estrogen deficiency. Moreover, DCQAs isolated from WSE were the main active compounds in WSE that exert anti-osteoclastogenic activity by regulating the RANKL-induced activation of NF-κB and MAPKs to inhibit ROS production. These results suggest that it may be possible to consume WSE as a health functional food for the purpose of preventing bone disease. The daily intake is expected to be within 800 mg/kg/day when converted by comparing the body surface area ratio of mice and humans [31]. Further studies are needed to confirm the safety of WSE ingestion by analyzing the concentrations of heavy metals, such as arsenic and mercury, and performing non-clinical research.

## Figures and Tables

**Table 1:** Primers used in this study.

Gene	Sequence (5′ to 3′)
*ACP5*	**F**: CTGGAGTGCACGATGCCAGCGACA
	**R**: TCCGTGCTCGGCGATGGACCAGA
*DCSTAMP*	**F**: CCAAGGAGTCGTCCATGATT
	**R**: GGCTGCTTTGATCGTTTCTC
*NFATc1*	**F**: GGGTCAGTGTGACCGAAGAT
	**R**: GGAAGTCAGAAGTGGGTGGA
*Ctsk*	**F**: GGCCAACTCAAGAAGA AAAC
	**R**: GTGCTTGCTTCCCTTCTGG
*β-actin*	**F**: AGGCCCAGAGCAAGAGAG
	**R**: TCAACATGATCTGGGTCATC

F: forward R: reverse.

## Data Availability

The data that support the findings of this study are available from the corresponding author upon reasonable request.

## Supplementary Materials

The following supporting information can be downloaded at: https://www.mdpi.com/article/10.3390/nu15234968/s1, Figure S1: Effect of ovariectomy and WSE on body weight and organ weight; Figure S2: UPLC-ESI-Q-TOF MS (negative) TIC chromatogram of WSE.; Figure S3: Ion chromatogram and ESI-MS spectrum of 3,4-dicaffeoylquinic acid; Figure S4: Ion chromatogram and ESI-MS spectrum of 3,5-dicaffeoylquinic acid; Figure S5: Ion chromatogram and ESI-MS spectrum of 4,5-dicaffeoylquinic acid; Figure S6: 1H NMR (500 MHz, CD3OD) spectrum of 3,4-dicaffeoylquinic acid; Figure S7: 13C NMR (125 MHz, CD3OD) spectrum of 3,4-dicaffeoylquinic acid; Figure S8: 1H-1H COSY spectrum of 3,4-dicaffeoylquinic acid; Figure S9: HSQC spectrum of 3,4-dicaffeoylquinic acid; Figure S10: HMBC spectrum of 3,4-dicaffeoylquinic acid; Figure S11: 1H NMR (500 MHz, CD3OD) spectrum of 3,5-dicaffeoylquinic acid; Figure S12: 13C NMR (125 MHz, CD3OD) spectrum of 3,5-dicaffeoylquinic acid; Figure S13: 1H-1H COSY spectrum of 3,5-dicaffeoylquinic acid; Figure S14: HSQC spectrum of 3,5-dicaffeoylquinic acid; Figure S15: HMBC spectrum of 3,5-dicaffeoylquinic acid; Figure S16: 1H NMR (500 MHz, CD3OD) spectrum of 4,5-dicaffeoylquinic acid; Figure S17: 13C NMR (125 MHz, CD3OD) spectrum of 4,5-dicaffeoylquinic acid; Figure S18: 1H-1H COSY spectrum of 4,5-dicaffeoylquinic acid; Figure S19: HSQC spectrum 4,5-dicaffeoylquinic acid; Figure S20: HMBC spectrum of 4,5-dicaffeoylquinic acid; Figure S21: WSE did not influence osteogenic differentiation of hUCB-MSC; Figure S22: Three DCQA derivatives from WSE suppressed RANKL-induced osteoclast differentiation; Figure S23: DCQA-containing fraction from WSE inhibited RANKL-stimulated activation of NF-B and MAPKs; Table S1: LC-ESI-MS data of WSE; Table S2: HR-ESI-MS (negative) data of three dicaffeoylquinic acids isolated from a water extract of desalted *Salicornia europaea*; Table S3: 1H NMR (500 MHz) data of three dicaffeoylquinic acids (isolated from a water extract of desalted *Salicornia europaea*) in CD3OD; Table S4: 13C NMR (125 MHz) data of three dicaffeoylquinic acids (isolated from a water extract of desalted *Salicornia europaea*) in CD3OD.
