# Supplementary material for: Water Extract of Desalted Salicornia europaea Inhibits RANKL-Induced Osteoclast Differentiation and Prevents Bone Loss in Ovariectomized Mice
Source: Nutrients. 2023 Nov 30;15(23):4968. doi: 10.3390/nu15234968 (PMC10708358; doi:10.3390/nu15234968)
Supplement: Supplementary file 1 [file nutrients-15-04968-s001.zip › nutrients-2712794-supplementary.pdf]

Water extract of desalted *Salicornia europaea* inhibits RANKL-induced osteoclast differentiation and prevents bone loss in ovariectomized mice

Ah-Ra Jang[a,b†], Yun-Ji Lee[a†], Dong-Yeon Kim[a], Tae-Sung Lee[a], Do-Hyeon Jung[a], Yeong-Jun Kim[a], In-Su Seo[a], Jae-Hun Ahn[a], Eun-Jung Song[b], Jisu Oh[b,c], Aoding Li[c], Si Hoon Song[c], Hyung-Sik Kim[d,e,f], Min-Jung Kang[d], Yoojin Seo[d], Jeong-Yong Cho[c*], Jong-Hwan Park[a,b*]

[a] Laboratory Animal Medicine, College of Veterinary Medicine and Animal Medical Institute, Chonnam National University, Gwangju 61186, Republic of Korea

[b] NODCURE, INC., 77 Yongbong-ro, Buk-gu, Gwangju 61186, Republic of Korea

[c] Department of Food Science & Technology, Chonnam National University, Gwangju 61186 Republic of Korea

[d] Department of Oral Biochemistry, Dental and Life Science Institute, School of Dentistry, Pusan National University, Yangsan 50612

[e] Department of Life Science in Dentistry, School of Dentistry, Pusan National University, Yangsan 50612

[f] Education and Research Team for Life Science on Dentistry, Pusan National University, Yangsan 50612, Korea

1. Changes in body weight and organ weight after ovariectomy

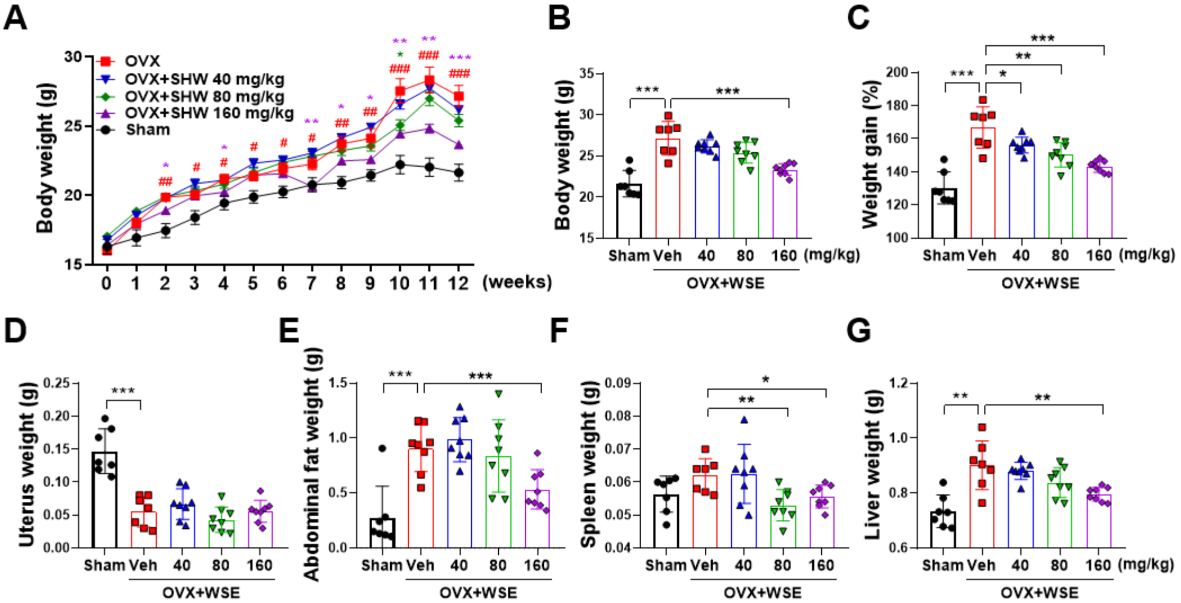

Figure S1. Effect of ovariectomy and WSE on body weight and organ weight

The mean body weight of the ovariectomized (OVX) group was significantly higher compared to that of the Sham group. The weights of the experimental animals were measured on a weekly basis. (A) The changes in body weight after OVX from 0 week to 12 weeks. (B) The body weight and (C) weight gain at 12 weeks after ovariectomy. The weights of (D) uterus, (E) abdominal fat, (F) spleen, and (G) liver at the end of the experiment.

2. LC-MS metabolomic analysis of WSE

The WSE metabolites were analyzed using an ultra-performance liquid chromatography−electrospray ionization hybrid ion-trap/time-of-flight mass spectrometer (UPLC−ESI−QTOF−MS) (SYNAPT G2; Waters, Cambridge, UK). Compounds were separated through an ACQUITY UPLC HSS T3 column (1.7 μm, 2.1 × 100 mm). Elution was performed using a gradient system of 100% H_2O containing 1% formic acid (eluent A) to 100% MeCN containing 1% formic acid (eluent B) with the following steps: 2% B for 2 min, 35% B for 7 min, 80% B for 13 min, and 100% B for 100 min. The mass conditions used were as

follows: capillary voltage, 3.5 kV; cone voltage, 40 V; source temperature, 120 °C; desolvation temperature, 500 °C; desolvation gas, 1050 L/h. Mass analysis was conducted in negative ionization mode using an electrospray ionization (ESI) source, and the collision energy was set at 25−50 eV.

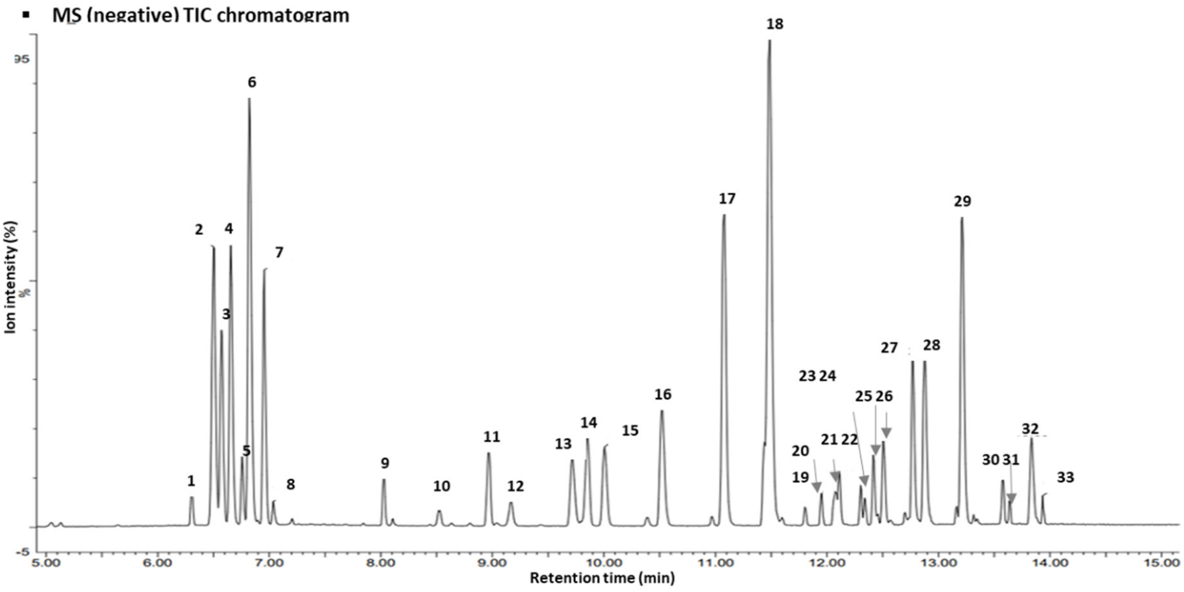

Figure S2. UPLC-ESI-Q-TOF MS (negative) TIC chromatogram of WSE

Table S1. LC-ESI-MS data of WSE

No.	Retention time (min)	Negative (*m/z*)	Molecular weight	Molecular formula	Expected compounds	RRF (%)
1	6.31	463.07	464	$C_{21}H_{20}O_{12}$	Quercetin glucoside	3.96
2	6.5	515.1	516	$C_{25}H_{24}O_{12}$	Dicaffeoyl quinic acid	44.59
3	6.57	517.12	518	$C_{25}H_{26}O_{12}$	Caffeoyldehydrocaffeoyl quinic acid	25.04
4	6.66	515.1	516	$C_{25}H_{24}O_{12}$	Dicaffeoyl quinic acid	37.74
5	6.7	477.09	478	$C_{22}H_{22}O_{12}$	Isorhamnetin glucoside	6.71
6	6.82	515.1	516	$C_{25}H_{24}O_{12}$	Dicaffeoyl quinic acid	63.55
7	6.96	517.12	518	$C_{25}H_{26}O_{12}$	Caffeoyldehydrocaffeoyl quinic acid	30.95
8	7.03	519.1	520	$C_{24}H_{24}O_{13}$	Isorhamnetin acetylglucoside	2.35
9	8.02	793.38	794	$C_{41}H_{62}O_{15}$	Norhederagenin glucuronide glucoside	5.8
10	8.53	791.36	792	$C_{41}H_{60}O_{15}$	Triterpene glucuronide glucoside	2.66
11	8.97	809.43	810	$C4_{2}H_{66}O_{15}$	Triterpene diglycoside	11.91
12	9.17	971.44	972	$C_{48}H_{76}O_{20}$	Triterpene triglycoside	4.6
13	9.71	941.41	942	$C_{47}H_{74}O_{19}$	Triterpene triglycoside	13.01
14	9.85	1087.5, 807.39	1088	$C_{53}H_{84}O_{23}$	Triterpene tetraglycoside	16.1
15	10.1	969.40, 849.40, 807.38	970	$C_{48}H_{74}O_{20}$	Triterpene triglycoside	15.26
16	10.5	939.39, 807.39	940	$C_{47}H_{72}O_{19}$	Triterpene triglycoside	24.61
17	11.08	955.42, 793.41	956	$C_{48}H_{76}O_{19}$	Triterpene triglycoside	58.35
18	11.49	925.41, 793.41, 631.33	926	$C_{46}H_{70}O_{19}$	Triterpene triglycoside	100
19	11.8	909.42	910	$C_{46}H_{70}O_{18}$	Triterpene triglycoside	2.04
20	11.95	939.42	940	$C_{47}H_{72}O_{19}$	Triterpene triglycoside	3.4
21	12.07	763.33, 629.31	764	$C_{39}H_{56}O_{15}$	Triterpene glycoside, tetrahydroxypentanoic acid	4.61
22	12.11	791.33, 671.33, 629.31	792	$C_{40}H_{56}O_{16}$	Triterpene glycoside	7.46
23	12.3	661.34	662	$C_{36}H_{54}O_{11}$	Triterpene glycoside	3.86
24	12.34	647.36	648	$C_{36}H_{56}O_{10}$	Triterpene glycoside, tetrahydroxypentanoic acid	2.7
25	12.42	809.37,	810	$C_{41}H_{62}O_{16}$	Triterpene glycoside,	7.55

		689.37, 647.36			tetrahydroxybutanoic acid	
26	12.51	761.32, 629.31	762	$C_{39}H_{54}O_{15}$	Triterpene glycoside, tetrahydroxypentanoic acid	10.4
27	12.76	807.35, 687.35, 645.35	808	$C_{41}H_{62}O_{16}$	Triterpene glycoside, tetrahydroxypentanoic acid	22.48
28	12.88	779.36, 647.36	780	$C_{40}H_{60}O_{15}$	Triterpene glycoside, tetrahydroxybutanoic acid	26.92
29	13.21	777.35, 645.35	778	$C_{40}H_{58}O_{15}$	Triterpene glycoside, tetrahydroxybutanoic acid	50.05
30	13.58	293.2	294	$C_{18}H_{30}O_{3}$	Oxyfattyacid	5.44
31	13.68	293.2	294	$C_{18}H_{30}O_{3}$	Oxyfattyacid	2.14
32	13.83	763.36, 631.36, 130.99	764	$C_{39}H_{56}O_{15}$	Triterpene glycoside, tetrahydroxybutanoic acid	14.11
33	13.93	295.22	296	$C_{18}H_{32}O_{3}$	Oxyfattyacid	1.87

Isolation of three DCQAs in WSE

Three DCQAs were purified and isolated from the WSE using ODS column chromatography. Briefly, medium-pressure liquid chromatography (Isolera one, Biotage, Uppsala, Sweden) was used to fractionate the WSE (1.1 g) through a Sfar C18 120-g column (flow rate, 50 mL/min; PDA detection, 254 and 220 nm). Elution was performed using a gradient system of 10% aqueous acetonitrile (MeCN) for 10 min, followed by 50% MeCN for 40 min to obtain DCQA fractions (15–20% MeCN eluate). The 15–20% MeCN eluate (150 mg) was separated through a Sfar C18 30-g column (flow rate, 20 mL/min; PDA detection, 254 and 220 nm) with step-wise elution of H_2O/MeCN (86:14, 84:16, 82:18, and 80:12 v/v; each step 300 mL) to obtain 3,4-DCQA (7.9 mg), 3,5-DCQA (11.2 mg), and 4,5-DCQA (11.0 mg).

3. MS and NMR results of three dicaffeoylquinic acids isolated from the WSE

Three dicaffeoylquinic acids (DCQAs) were analyzed using an ultra-performance liquid chromatography−electrospray ionization hybrid ion-trap/time-of-flight mass spectrometer (UPLC−ESI−QTOF−MS) (SYNAPT G2) with the same method described above. The DCQAs that were isolated in this study were dissolved in deuterated methanol (CD_3OD; Merck Co., Darmstadt, Germany). A ^{unity}INOVA 600 spectrometer (Varian, Walnut Creek, CA, USA; Korean Basic Science Institute Gwangju Center) was used to obtain ^1H and ^{13}C nuclear magnetic resonance (NMR) spectra. Connections between the caffeic acid and quinic acid were assigned using 2D-NMR experiments, including homonuclear correlation spectroscopy (^1H-^1H COSY), heteronuclear single quantum coherence (HSQC), and heteronuclear multiple bond correlation (HMBC).

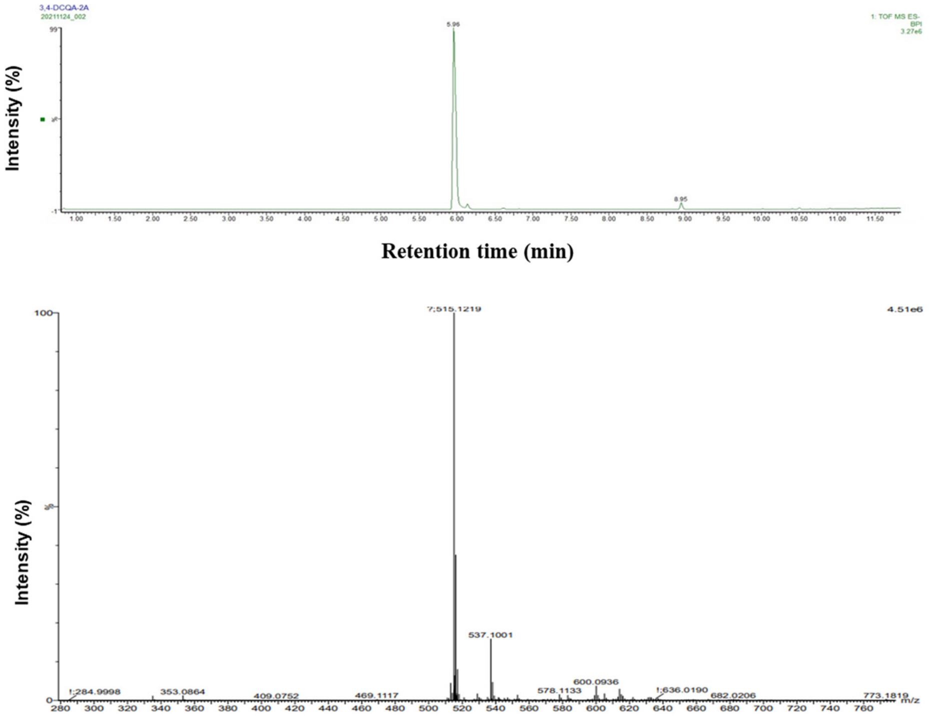

Figure S3. Ion chromatogram and ESI-MS spectrum of 3,4-dicaffeoylquinic acid

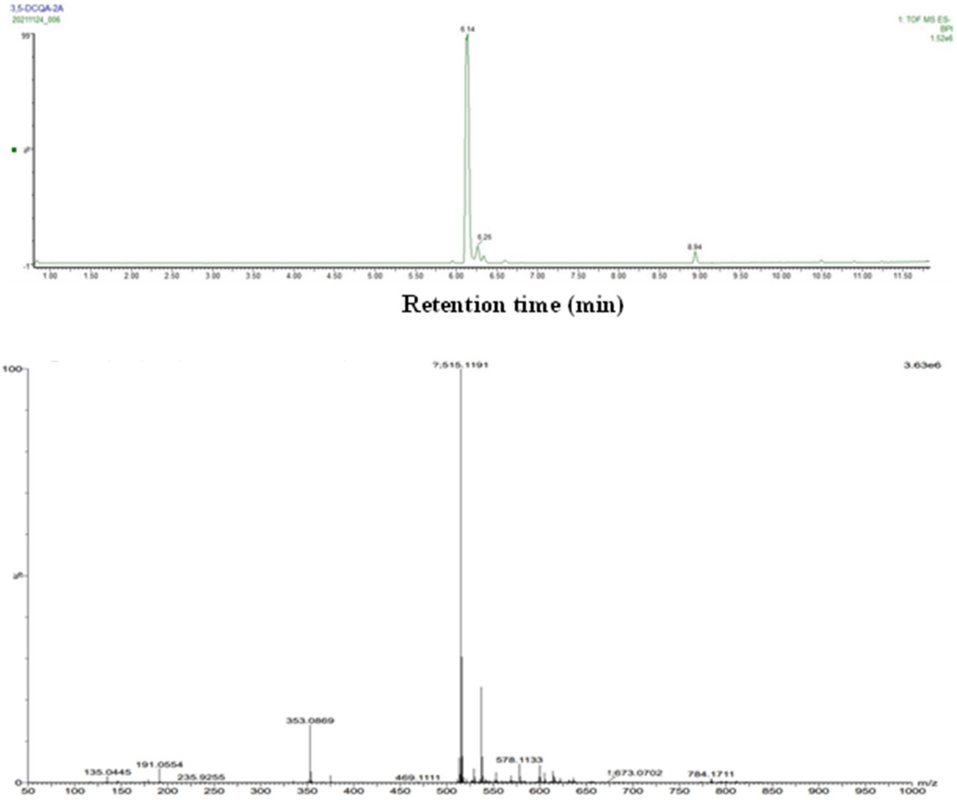

Figure S4. Ion chromatogram and ESI-MS spectrum of 3,5-dicaffeoylquinic acid

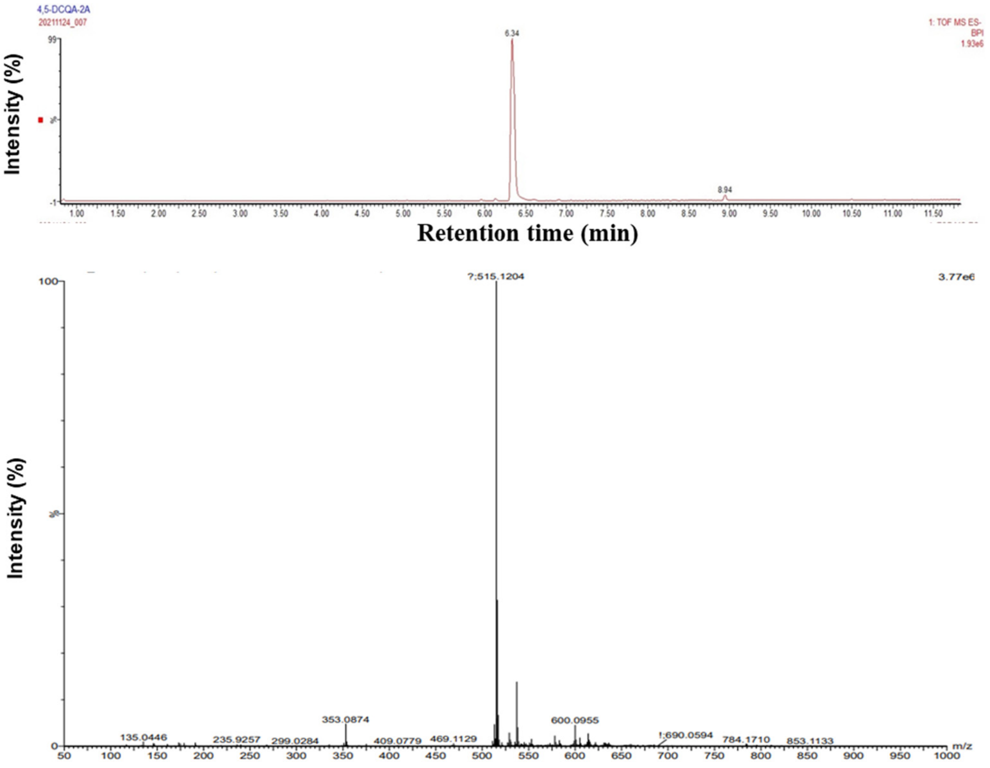

Figure S5. Ion chromatogram and ESI-MS spectrum of 4,5-dicaffeoylquinic acid

Table S2. HR-ESI-MS (negative) data of three dicaffeoylquinic acids isolated from a water extract of desalted *Salicornia europaea*

Compounds	Analytic mass (*m/z*, $[M-H]^-$)	Calc. mass (*m/z*, $[M-H]^-$)	mDa	Molecular formula
3,4-DCQA	515.1187	515.1190	-0.3	$C_{25}H_{23}O_{12}$
3,5-DCQA	515.1290	515.1190	0.0	$C_{25}H_{23}O_{12}$
4,5-DCQA	515.1291	515.1190	+0.1	$C_{25}H_{23}O_{12}$

DCQA, dicaffeoylquinic acid.

Table S3. ^{1}H NMR (500 MHz) data of three dicaffeoylquinic acids (isolated from a water extract of desalted *Salicornia europaea*) in CD_3OD

Position	δ_H (*Int.*, *Multi.*, *J* in Hz)		
	**3,4-Dicaffeoylquinic acid**	**3,5-Dicaffeoylquinic acid**	**4,5-Dicaffeoylquinic acid**
2a	2.32 (1H, m)	2.20 (2H, m)	2.20 (1H, br. d, 5.4)
2b	2.15 (1H, br. d, 4.0)		2.13 (1H, m)
3	5.42 (1H, m)	5.42 (1H, m)[a]	4.36 (1H, m)
4	5.03 (1H, dd, 3.0, 3.0)	3.95 (1H, dd, 3.0, 3.0)	5.12 (1H, br. d, 8.5)
5	4.32 (1H, m)	5.42 (1H, m)[a]	5.67 (1H, br. s)
6a	2.20 (1H, m)	2.32 (1H, m)	2.32 (1H, br. d, 12.5)
6b	2.13 (1H, m)	2.15 (1H, m)	2.15 (1H, br. d, 4.0)
2'	7.03 (1H, d, 2.0)	7.07 (2H, d, 1.8)	6.98 (1H, br. s)
5'	6.78 (1H, d, 8.0)	6.79 (1H, s)	6.75 (1H, d, 8.0)
6'	6.88 (1H, dd, 8.0, 2.0)	6.97 (1H, t)	6.91 (1H, br. d, 8.0)
7'	7.55 (1H d, 16.0)	7.58 (1H d, 15.6)	7.58 (1H d, 16.0)
8'	6.28 (1H, d, 16.0)	6.37 (1H, d 15.6)	6.32 (1H, d, 16.0)
2''	7.03 (1H, d, 2.0)	7.06 (2H, d, 1.8)	7.01 (1H, br. s)
5''	6.78 (1H, d, 8.0)	6.78 (1H, s)	6.74 (1H, br. d, 8.0)
6''	6.92 (1H, br. d, 8.0)	6.96 (1H, t)	6.88 (1H, br. d, 8.0)
7''	7.56 (1H, d, 16.0)	7.62 (1H, d, 15.6)	7.50 (1H, d, 15.6)
8''	6.26 (1H, d, 16.0)	6.28 (1H, d, 15.6)	6.28 (1H, d, 15.6)

[a]Signals of H-3 and H-5 overlapped.

Table S4. ^{13}C **NMR (125 MHz) data of three dicaffeoylquinic acids (isolated from a water extract of desalted *Salicornia europaea*) in** CD_3OD

Position	δ_C		
	3,4-Dicaffeoylquinic acid	3,5-Dicaffeoylquinic acid	4,5-Dicaffeoylquinic acid
1	74.3	75.4	71.5
2	41.6	38.7	38.7
3	70.2	73.3	69.5
4	76.2	71.5	76.7
5	66.3	72.4	70.4
6	37.3	36.6	40.3
7	178.6	178.6	178.6
1'	127.9	128.1	128.1
2'	115.3	115.3	115.3
3'	146.9	146.9	146.9
4'	147.5	149.7	149.7
5'	116.6	116.6	116.6
6'	123.4	123.2	123.2
7'	149.8	147.3	147.3
8'	115.4	115.4	115.4
9'	168.6	169.2	169.2
1''	127.9	128.0	128.0
2''	115.1	115.3	115.3
3''	146.9	146.9	146.9
4''	147.5	149.6	149.6
5''	116.6	116.6	116.6
6''	123.2	123.1	123.1
7''	149.7	147.1	147.1
8''	115.1	115.4	115.4
9''	168.7	168.7	168.7

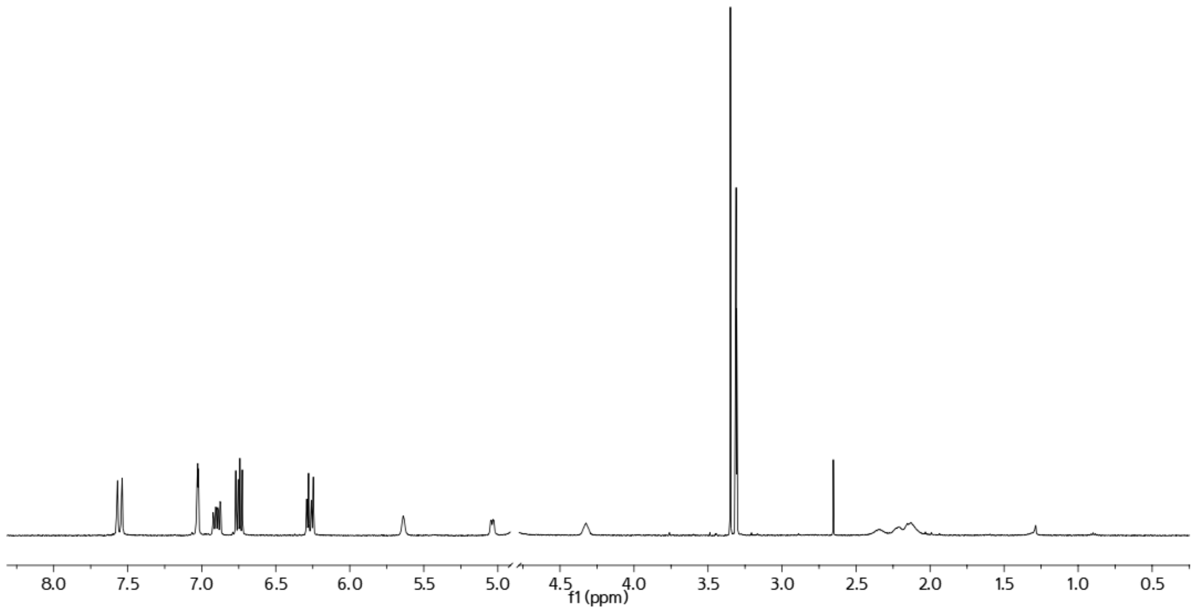

Figure S6. ^{1}H NMR (500 MHz, CD_3OD) spectrum of 3,4-dicaffeoylquinic acid

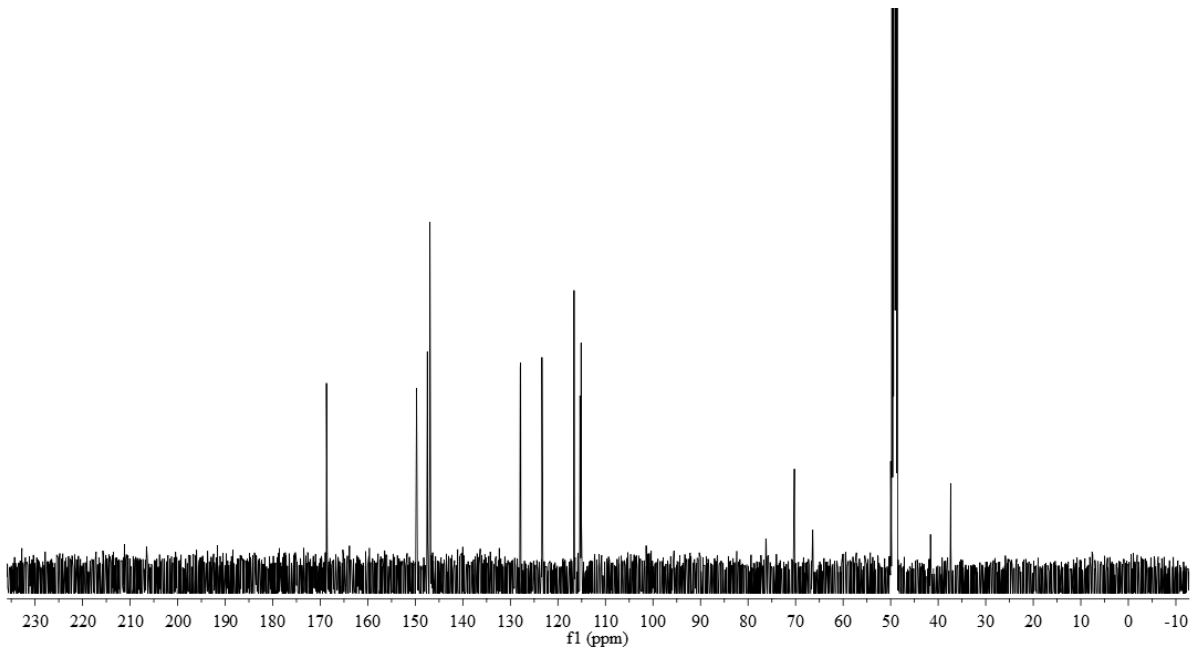

Figure S7. ^{13}C NMR (125 MHz, CD_3OD) spectrum of 3,4-dicaffeoylquinic acid

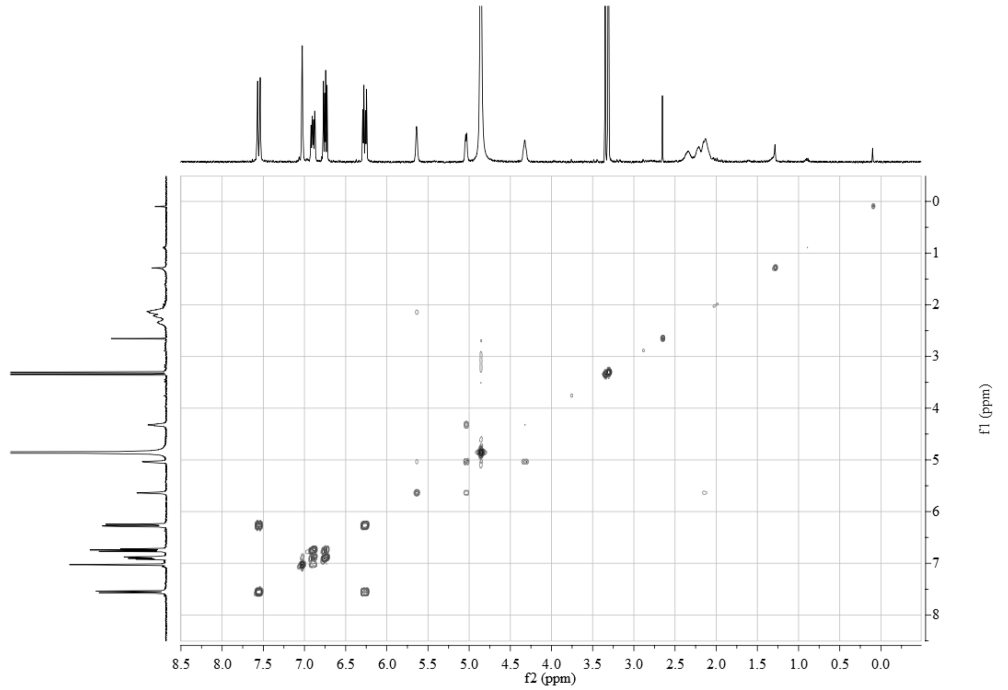

Figure S8. ^{1}H-^{1}H COSY spectrum of 3,4-dicaffeoylquinic acid

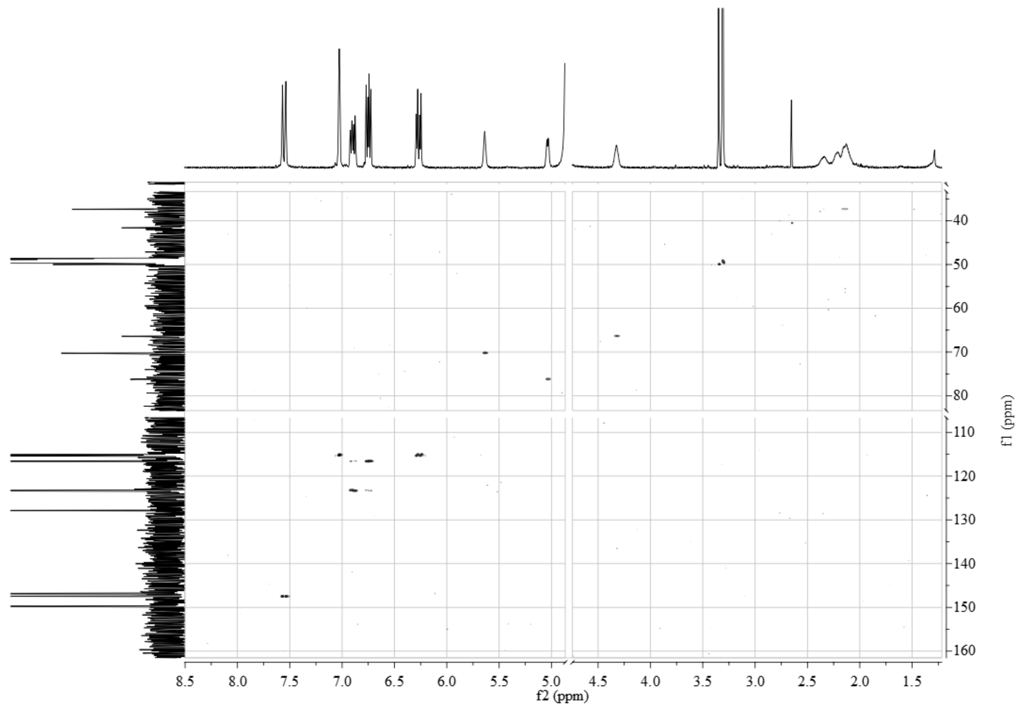

Figure S9. HSQC spectrum of 3,4-dicaffeoylquinic acid

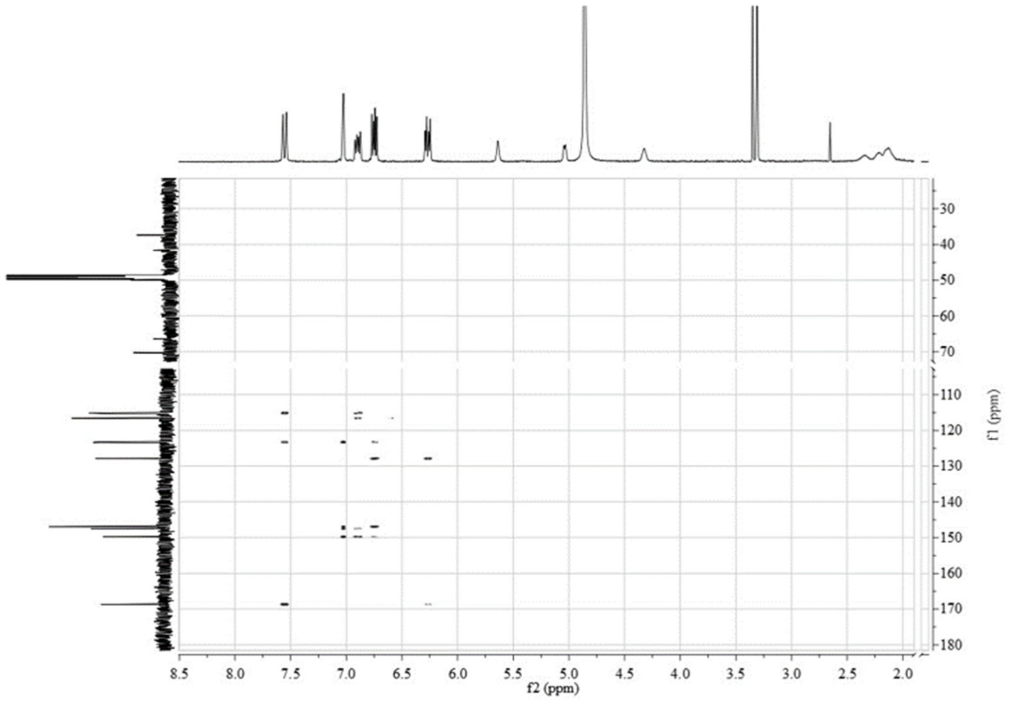

Figure S10. HMBC spectrum of 3,4-dicaffeoylquinic acid

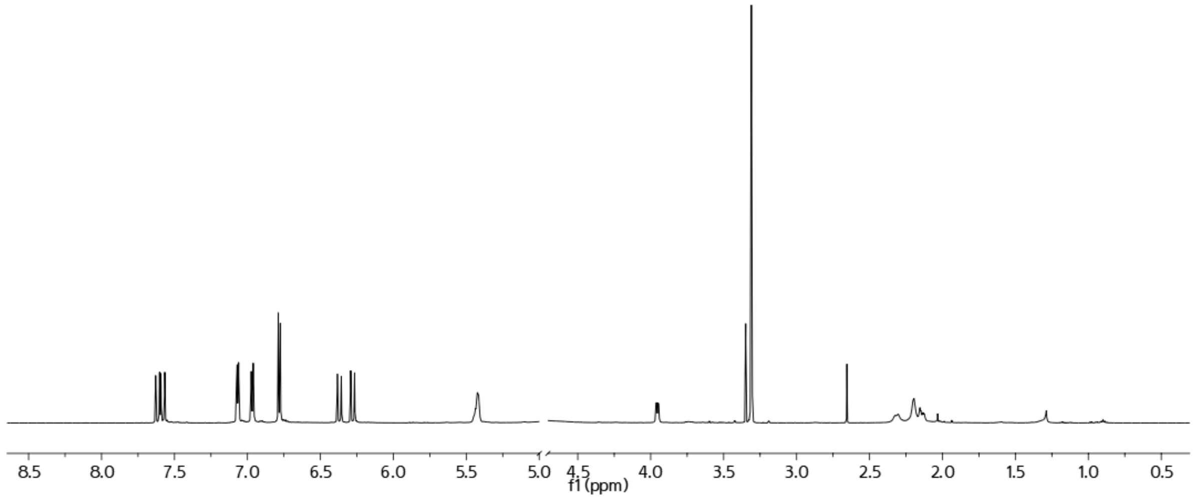

Figure S11. ^{1}H NMR (500 MHz, CD_3OD) spectrum of 3,5-dicaffeoylquinic acid

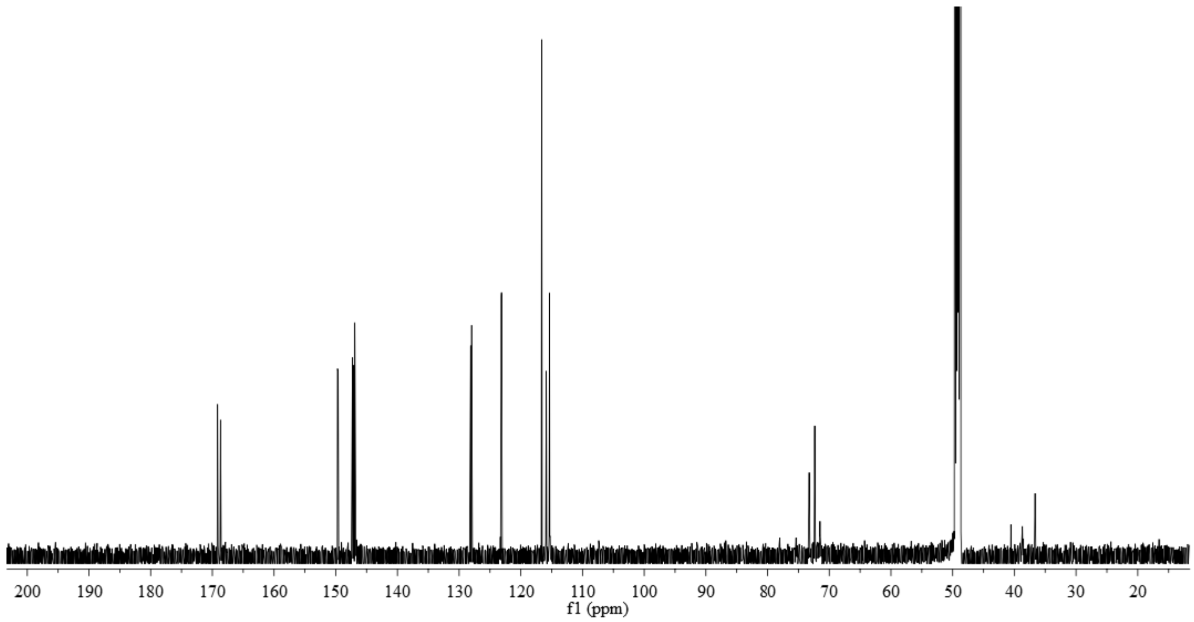

Figure S12. ^{13}C **NMR (125 MHz,** CD_3OD**) spectrum of 3,5-dicaffeoylquinic acid**

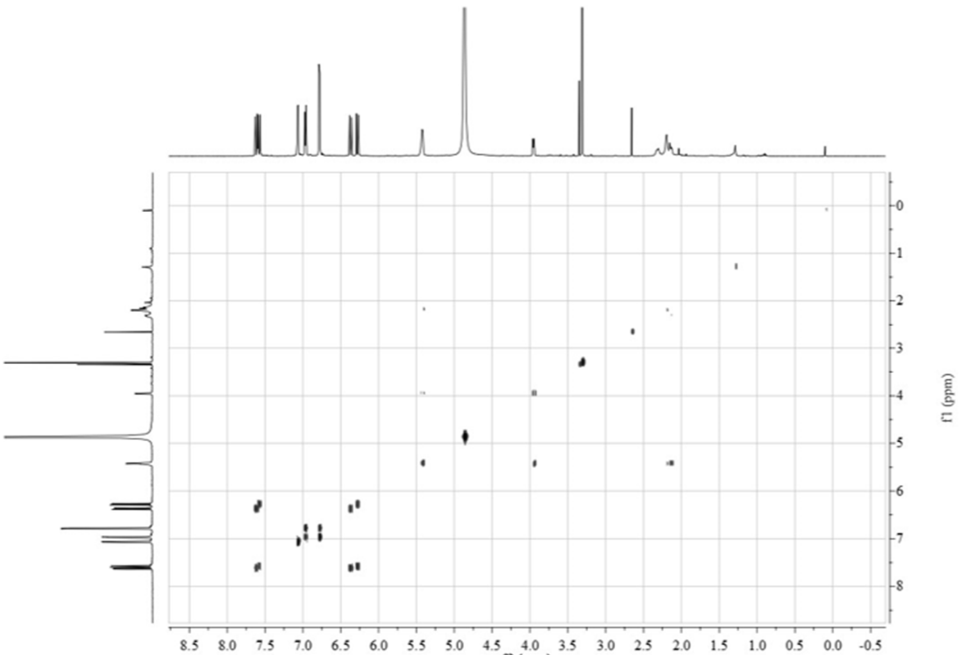

Figure S13. ^1H-^1H **COSY spectrum of 3,5-dicaffeoylquinic acid**

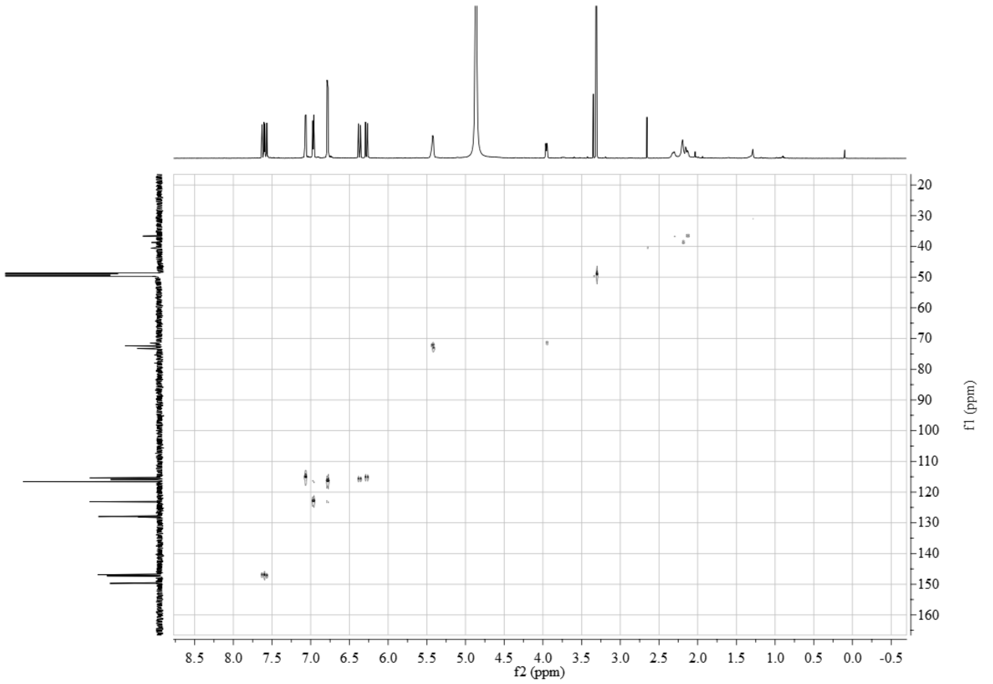

Figure S14. HSQC spectrum of 3,5-dicaffeoylquinic acid

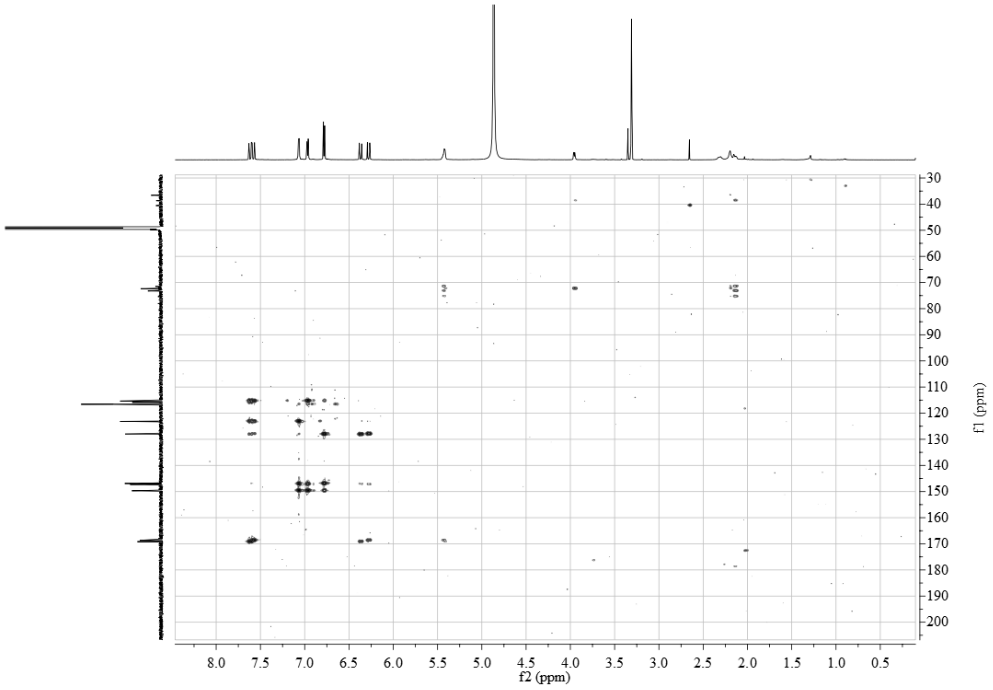

Figure S15. HMBC spectrum of 3,5-dicaffeoylquinic acid

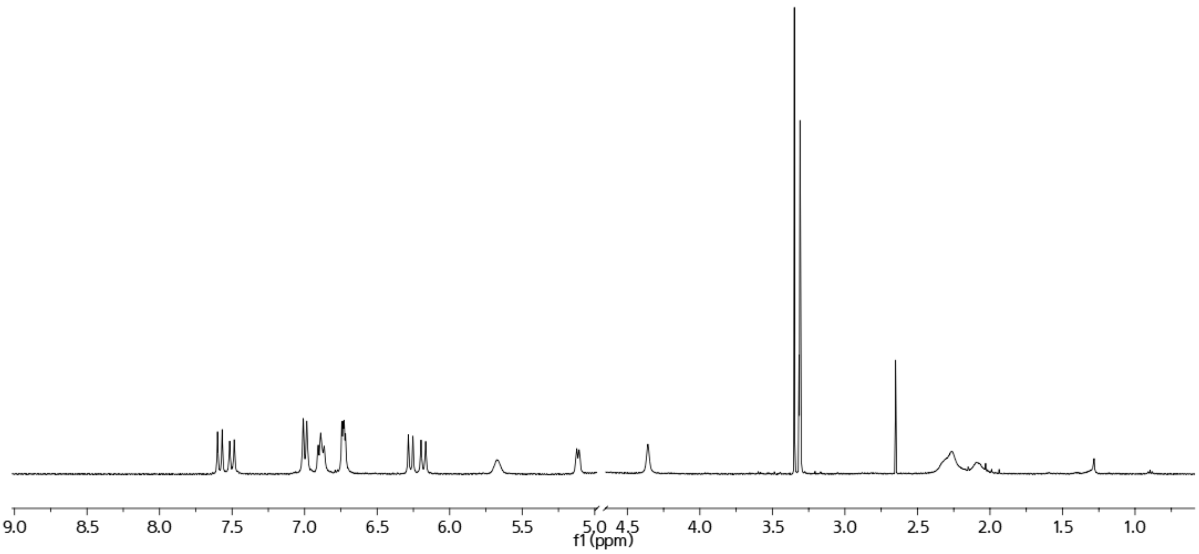

Figure S16. ^{1}H NMR (500 MHz, CD_3OD) spectrum of 4,5-dicaffeoylquinic acid

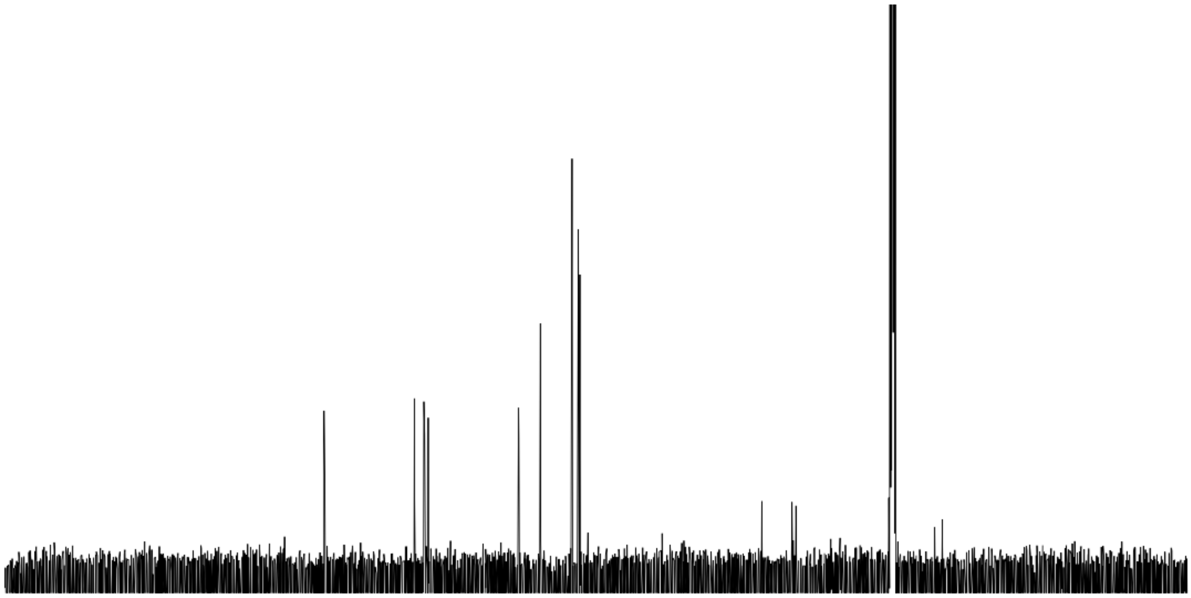

Figure S17. ^{13}C NMR (125 MHz, CD_3OD) spectrum of 4,5-dicaffeoylquinic acid

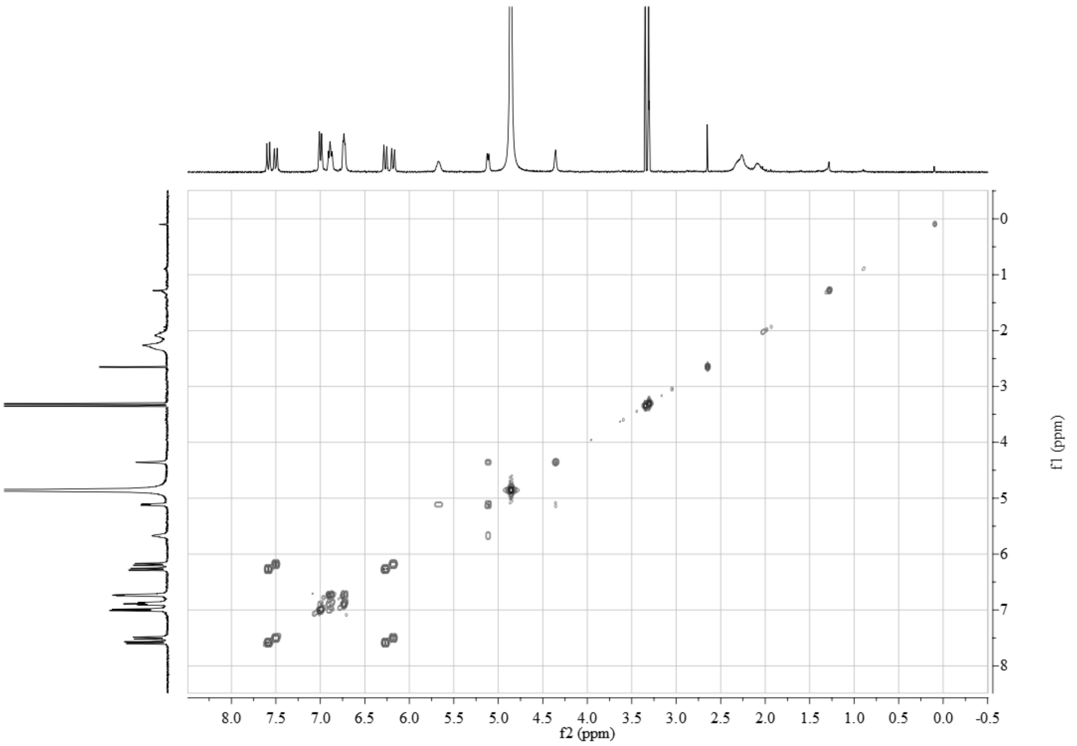

Figure S18. ^{1}H-^{1}H COSY spectrum of 4,5-dicaffeoylquinic acid

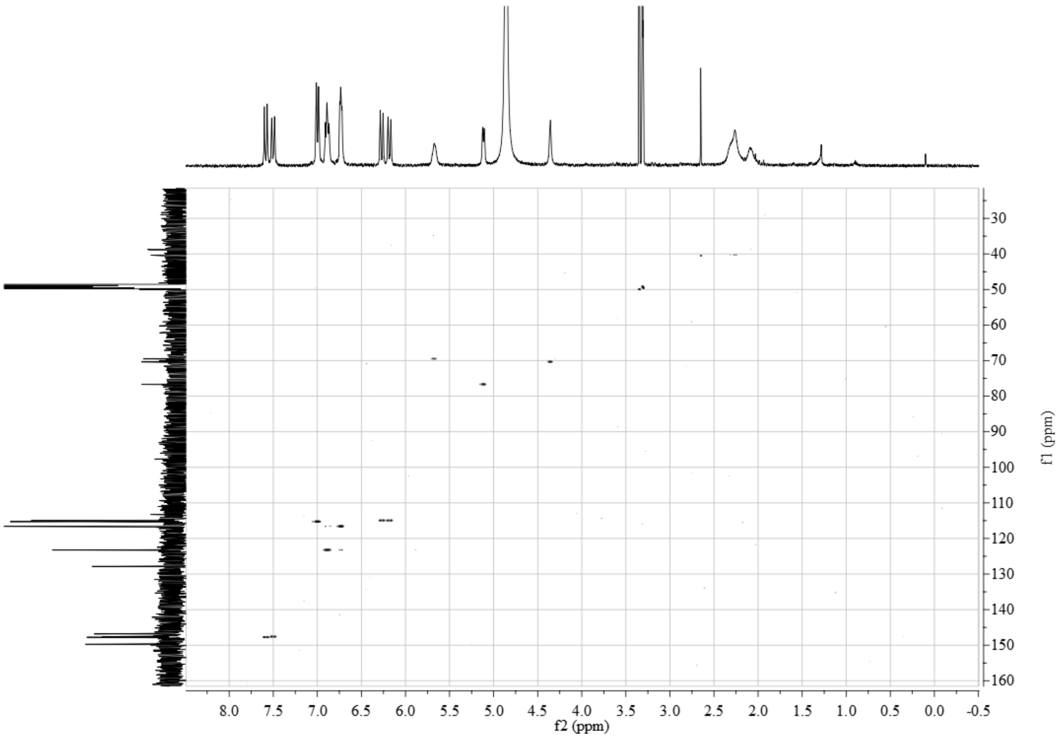

Figure S19. HSQC spectrum 4,5-dicaffeoylquinic acid

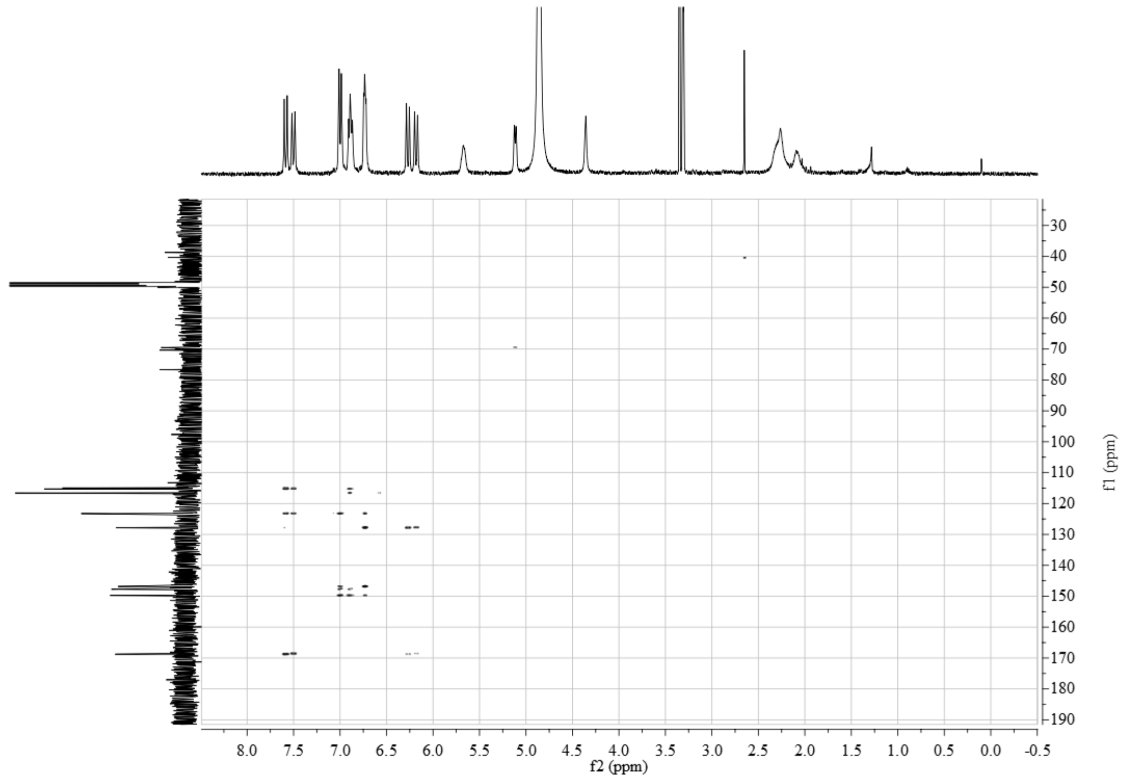

Figure S20. HMBC spectrum of 4,5-dicaffeoylquinic acid

4. Assessment of Osteoblast Differentiation Levels Induced by WSE

Human UCB-MSCs culture

Human umbilical cord blood-derived mesenchymal stem cells (hUCB-MSCs) were obtained from Kangstem Biotech (Seoul, Republic of Korea). The hUCB-MSCs were cultured in KSB-3 basal medium supplemented with KSB-3 supplements (Kangstem Biotech, Seoul, Republic of Korea), 10% fetal bovine serum (FBS; Gibco, Grand Island) and 100 U/ml penicillin/streptomycin (Gibco) at 37 ℃ with 5% CO_2.

Osteogenic differentiation

Human UCB-MSCs were seeded in 6 well plates at a density of 1.5×10^5 cells/well in KSB-3 medium. When the confluency reached 40–50%, the medium was changed to high-glucose DMEM containing 10% FBS, 0.1 μM dexamethasone, and 10 mM beta-glycerophosphate (Sigma) to initiate osteogenic differentiation. Cells were incubated in the presence of WSE-containing osteogenic medium for 1 or 2 weeks with medium changes every 3 days. After 1 or 2 weeks of induction, cells were fixed with 70% ethanol at 4 °C for 20 minutes and stained with Alizarin red S (Kanto, Tokyo, Japan) at room temperature for 10 minutes. The staining solution was aspirated and then cells were washed with distilled water. The quantity of staining was observed using an optical microscope, and when the dye was completely eluted by adding 10% cetylpyridinium chloride (Sigma), the staining was quantified using a spectrophotometer at 570 nm.

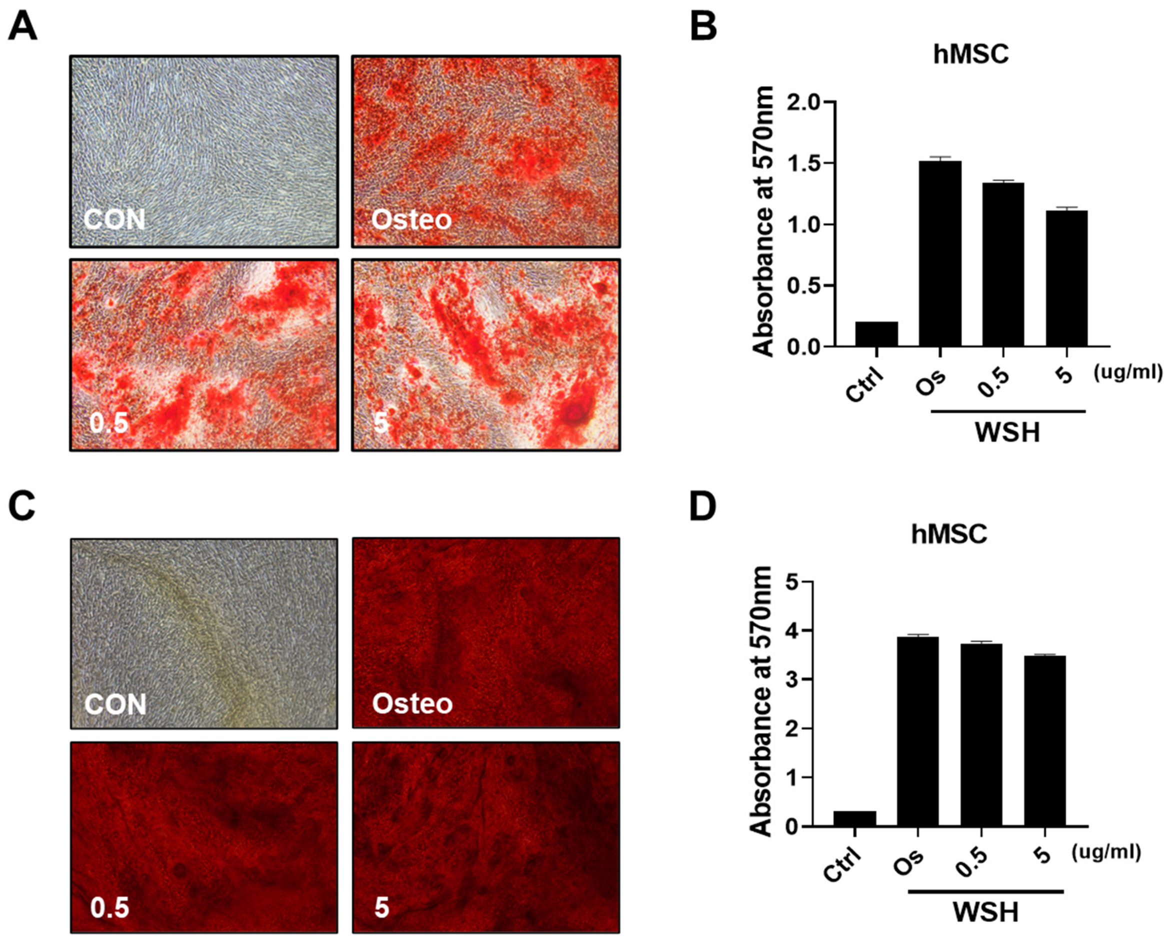

Figure S21. WSE did not influence osteogenic differentiation of hUCB-MSCs.

Human UCB-MSCs were cultured in conditioned media with or without WSE (0.5 and 5 μg/ml) for either 1 (A and B) or 2 weeks (C and D). After osteogenic differentiation, the cells were fixed, stained with Alizarin red S, and the optical density was measured for quantification using a spectrophotometer at 570 nm.

5. Three DCQAs (isolated from a water extract of desalted *Salicornia europaea*) suppressed RANKL-induced osteoclast differentiation.

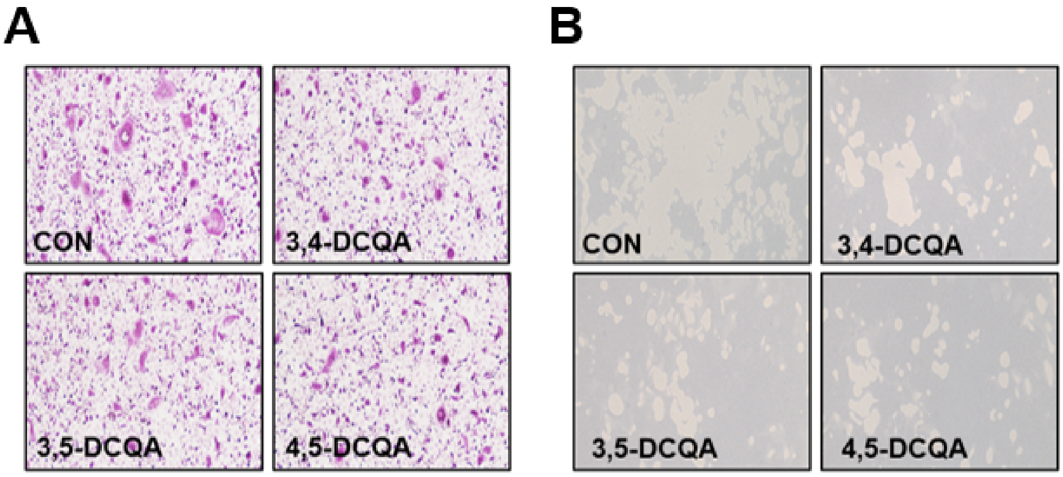

Figure S22. Three DCQA derivatives from WSE suppressed RANKL-induced osteoclast differentiation

BMDMs were pretreated with three types of DCQAs (10 μM): 3,4-DCQA, 3,5-DCQA, and 4,5-DCQA. These were extracted from WSE for 2 h and subsequently stimulated with RANKL for 3 days. (A) RANKL-induced osteoclasts were fixed and stained to detect TRAP activity. (B) BMDMs were seeded on an Osteo Assay Surface plate for 7 days, then cells were removed, and resorption pits were imaged.

6. DCQA-containing fraction from WSE inhibited RANKL-stimulated activation of NF-κB and MAPKs.

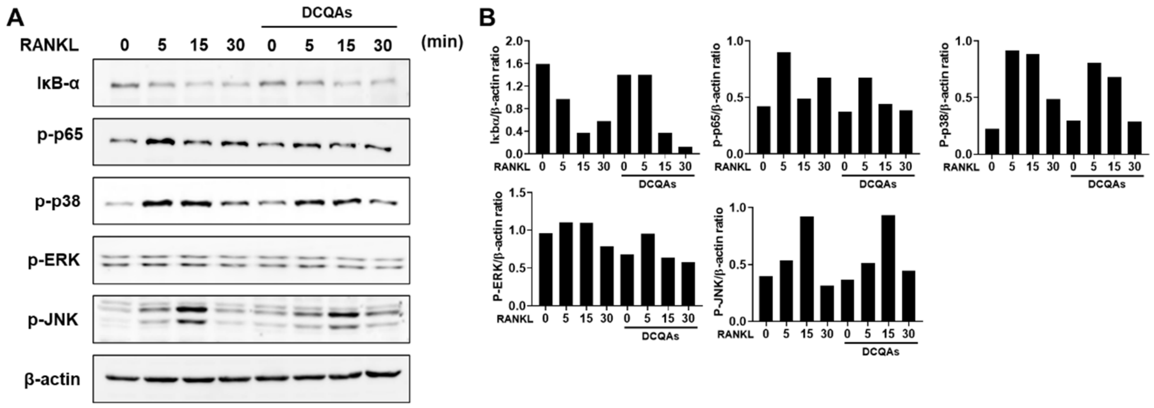

Figure S23. DCQA-containing fraction from WSE inhibited RANKL-stimulated activation of NF-κB and MAPKs.

BMDMs were pretreated with DCQA fractions for 2 h and subsequently stimulated with RANKL for the indicated time points (A and B). Cell lysates were extracted and analyzed for IκB-α degradation, as well as phosphorylation of p65, p38, ERK, and JNK, using western blotting. An antibody against β-actin was used to confirm the loading doses.
